# Normal sex and age-specific parameters in a multi-ethnic population: a cardiovascular magnetic resonance study of the Canadian Alliance for Healthy Hearts and Minds cohort

**DOI:** 10.1186/s12968-021-00819-z

**Published:** 2022-01-03

**Authors:** Judy M. Luu, Catherine Gebhard, Chinthanie Ramasundarahettige, Dipika Desai, Karleen Schulze, Francois Marcotte, Philip Awadalla, Philippe Broet, Trevor Dummer, Jason Hicks, Eric Larose, Alan Moody, Eric E. Smith, Jean-Claude Tardif, Tiago Teixeira, Koon K. Teo, Jennifer Vena, Douglas S. Lee, Sonia S. Anand, Matthias G. Friedrich, the CAHHM Study Investigators

**Affiliations:** 1 Division of Cardiology, Department of Medicine, University of Manitoba, Winnipeg, MB Canada; 2 Department of Nuclear Medicine, University Hospital Zurich, Zurich, Switzerland; 3 Center for Molecular Cardiology, University of Zurich, Zurich, Switzerland; 4 Population Health Research Institute, Hamilton Health Sciences, McMaster University, 237 Barton St East, Hamilton, ON L8L 2X2 Canada; 5 Department of Medicine, McMaster University, 1280 Main Street West, Hamilton, ON L8S 4K1 Canada; 6 Research Centre, Montreal Heart Institute, Université de Montréal, 5000 Belanger Street, Montreal, QC H1T 1C8 Canada; 7 Department of Molecular Genetics, Ontario Institute for Cancer Research, University of Toronto, 661 University Avenue Suite 510, Toronto, ON M5G 0A3 Canada; 8 Department of Preventive and Social Medicine, École de Santé Publique, Université de Montréal, 3175 Chemin de la Cote-Sainte-Catherine, Montreal, QC H3T 1C5 Canada; 9 Research Centre, CHU Sainte Justine, 3175 Chemin de la Cote-Sainte-Catherine, Montreal, QC H3T 1C5 Canada; 10 School of Population and Public Health, Cancer Control Research, BC Cancer, University of British Columbia, 675 W 10th Avenue, Vancouver, BC V5Z 1L3 Canada; 11 Atlantic PATH, Dalhousie University, 1494 Carlton Street, P.O. Box 15000, Halifax, NS B3H 4R2 Canada; 12 Institut Universitaire de Cardiologie et de Pneumologie de Québec - Université Laval, 2725 chemin Sainte-Foy, Quebec, G1V 4G5 Canada; 13 Sunnybrook Health Science Centre, Toronto, ON Canada; 14 Department of Clinical Neurosciences, Hotchkiss Brain Institute, University of Calgary, 3330 Hospital Drive NW, Calgary, AB T2N 4N1 Canada; 15 Cardiology Department, Entre Douro e Vouga Hospital Centre, Santa Maria Feira, Portugal; 16 Department of Health Evidence and Impact, McMaster University, 1280 Main Street West, Hamilton, ON L8S 4K1 Canada; 17 Cancer Research and Analytics, Cancer Control Alberta, Alberta Health Services, Suite 1500 Sun Life Place, 10123 99th Street NW, Edmonton, AB T5J 3H1 Canada; 18 Institute for Clinical Evaluative Sciences, Toronto, ON Canada; 19 Peter Munk Cardiac Centre University Health Network University of Toronto, Toronto, ON Canada; 20 Department of Medicine and Diagnostic Radiology, McGill University, 1001 Decarie Boulevard, Montreal, QC H4A 3J1 Canada

**Keywords:** Sex, Age, Cardiovascular magnetic resonance, Reference, Normal

## Abstract

**Background:**

Despite the growing utility of cardiovascular magnetic resonance (CMR) for cardiac morphology and function, sex and age-specific normal reference values derived from large, multi-ethnic data sets are lacking. Furthermore, most available studies use a simplified tracing methodology. Using a large cohort of participants without history of cardiovascular disease (CVD) or risk factors from the Canadian Alliance for Healthy Heart and Minds, we sought to establish a robust set of reference values for ventricular and atrial parameters using an anatomically correct contouring method, and to determine the influence of age and sex on ventricular parameters.

**Methods and results:**

Participants (n = 3206, 65% females; age 55.2 ± 8.4 years for females and 55.1 ± 8.8 years for men) underwent CMR using standard methods for quantitative measurements of cardiac parameters. Normal ventricular and atrial reference values are provided: (1) for males and females, (2) stratified by four age categories, and (3) for different races/ethnicities. Values are reported as absolute, indexed to body surface area, or height. Ventricular volumes and mass were significantly larger for males than females (p < 0.001). Ventricular ejection fraction was significantly diminished in males as compared to females (p < 0.001). Indexed left ventricular (LV) end-systolic, end-diastolic volumes, mass and right ventricular (RV) parameters significantly decreased as age increased for both sexes (p < 0.001). For females, but not men, mean LV and RVEF significantly increased with age (p < 0.001).

**Conclusion:**

Using anatomically correct contouring methodology, we provide accurate sex and age-specific normal reference values for CMR parameters derived from the largest, multi-ethnic population free of CVD to date.

**Clinical trial registration:**

ClinicalTrials.gov, NCT02220582. Registered 20 August 2014—Retrospectively registered, https://clinicaltrials.gov/ct2/show/NCT02220582.

**Supplementary Information:**

The online version contains supplementary material available at 10.1186/s12968-021-00819-z.

## Introduction

Over the last several years, cardiovascular magnetic resonance (CMR) imaging has been established as a reproducible reference standard for the quantification of chamber volumes, function, and mass in the evaluation of various cardiac diseases [1]. Despite growing awareness of sex/gender and age-related differences in diagnostic approaches [2], CMR-based reference values for ventricular and atrial parameters, specific to sex and age groups are lacking, with conflicting data derived from small or heterogeneous populations [3–11]. Furthermore, previously available reference values were limited by sample cohorts with established cardiovascular disease (CVD) risk factors, discrete ethnic populations [10, 12–14], or by a contouring methodology that excluded trabecular tissue and papillary muscle from left ventricular (LV) mass, thus was not anatomically accurate [15].

A large prospective, multi-center cohort through the Canadian Alliance for Healthy Heart and Minds (CAHHM) provides a unique opportunity to investigate normal ranges of physiologic parameters, as well as the association with socio-environmental and contextual factors, CVD risk, subclinical disease, and other related chronic disease outcomes [16]. In addition to extensive clinical assessments consisting of health questionnaires, physical measurements, and blood sample collection, CAHHM participants also underwent a comprehensive magnetic resonance imaging (MRI) of the brain, heart, carotid artery, and abdomen [16]. A total of 8,580 participants were recruited with the opportunity to better understand the data of those who completed CMR imaging.

Given the widespread use and the incremental prognostic value of CMR, the objectives of this study were to establish a robust set of reference values for ventricular/atrial volumetric and functional parameters, and to understand the relationship with age and sex in participants without a history of established CVD or CVD risk factors.

## Methods

### Study population

The CAHHM is a prospective study of participants recruited through existing research cohorts, with each separate inclusion and exclusion criteria, as previously described [16]. Research ethics approval was granted by the Hamilton Integrated Research Ethics Board, with consent obtained at each collaborating site as per site-specific regulations prior to participation in the study. Selection criteria specific to CAHHM included males and females, between the ages of 35 and 75 years, who were willing to undergo an MRI scan and all other required study procedures. A parallel Alliance-First Nations cohort was also undertaken in partnership with eight First Nations communities, however, the data was not included in this sub-study. Balanced representation of participants across different age strata 35–44, 44–54, 55–64, and 65–74 years with approximately 50% or more recruitment of females was also ensured [16]. Additional file 1: Figure S1 shows the flowchart (for participant selection and recruitment). For the purposes of this analysis, participants with CVD defined as a clinical history of angina or myocardial infarction, stroke, heart failure and/or other cardiac disease, prior coronary artery bypass grafting, or percutaneous coronary intervention were excluded. Participants with hypertension defined as a resting elevated blood pressure > 140/80 mmHg, diabetes, obesity (body mass index (BMI) > 30), smoking, or dyslipidemia were also excluded. Finally, participants were ineligible for recruitment if they had contraindications to CMR, including claustrophobia, pregnancy, non-compatible pacemaker/defibrillator devices, and intraocular/intracranial metallic materials. This study complied with the STROBE (Strengthening the Reporting of Observational Studies in Epidemiology) checklist.

### CMR protocol

CMR images were acquired at collaborating sites with the participants in supine position using conventional CMR systems (1.5 T or 3 T) and a cardiac (preferred) or chest phased-array surface coil with ≥ 8 receiver elements. Following appropriate magnetic shimming, standard localization was performed with a single-breath hold, retrospective electrocardiogram (ECG)-gated balanced steady state free precession (bSSFP) sequence. When available, bSSFP frequency scouting was performed to minimize susceptibility artifacts in the myocardium. Cine images were acquired for LV and right ventricular (RV) volumetric and functional parameters in both the long axis (2 slices) and contiguous short axis (SAx) views (12–14 slices). Field of view was 360 mm, voxel size ranged from 0.9 to 1.2 mm, while slice thickness was 8 mm, with a 2 mm gap.

### Image analysis

**Fig. 1:**
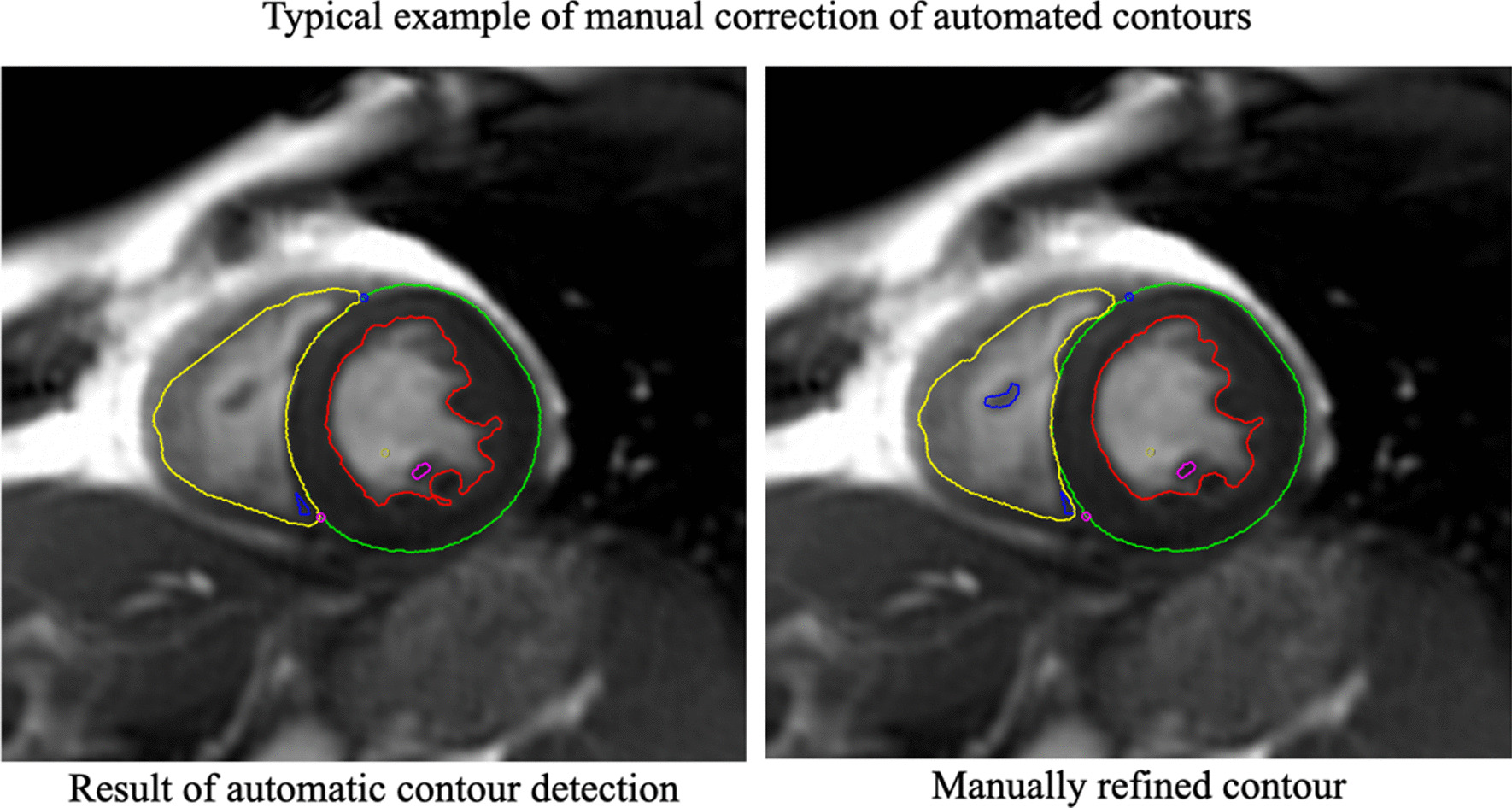
A typical example of an automatically generated contour (left) and the result of manual correction (right)

**Fig. 2:**
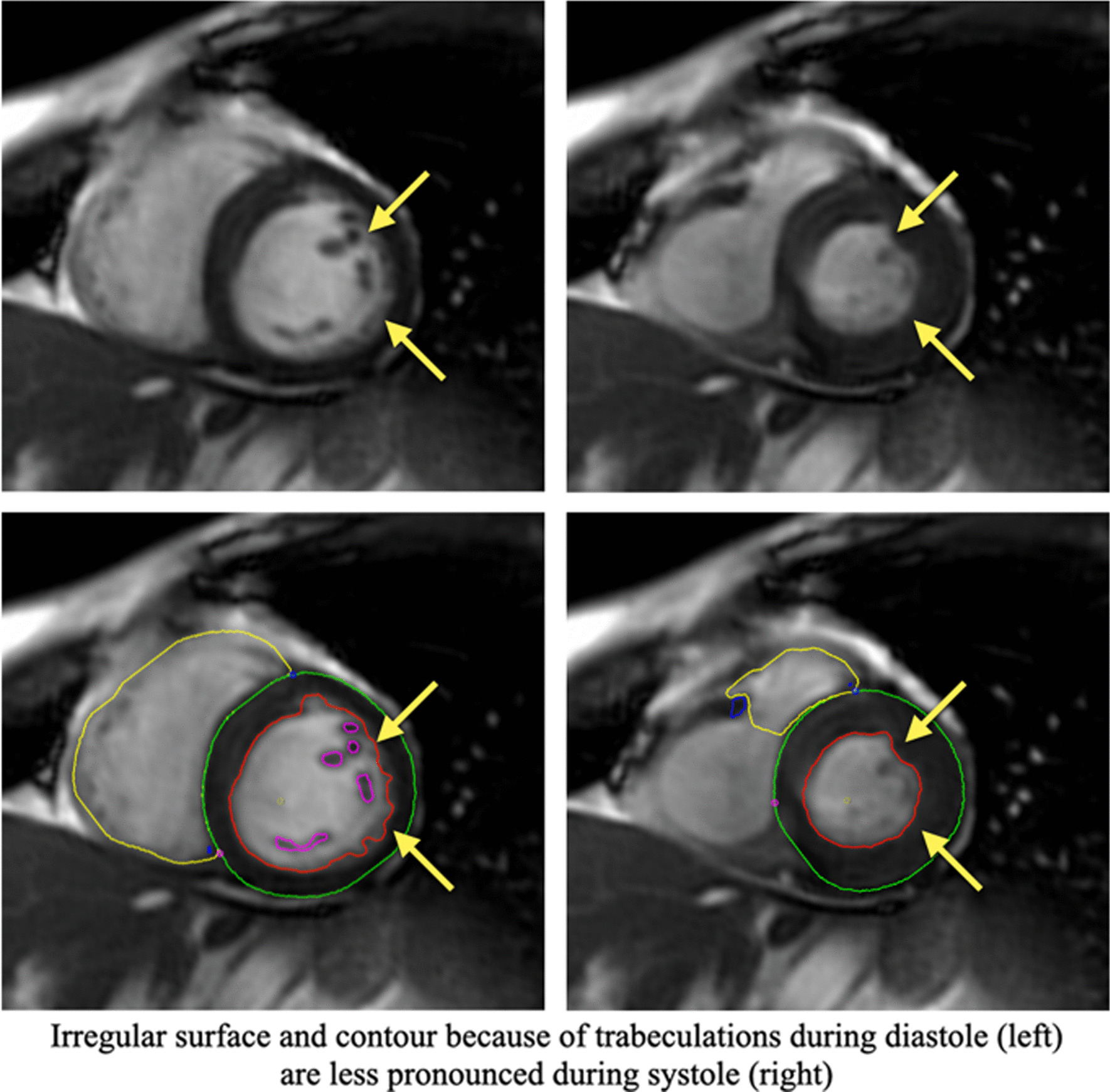
Measurement of left ventricular (LV) mass at end-systole. The systolic phase is used for quantification of LV mass; irregular surface and contour because of trabeculations during diastole (left) are less pronounced during systole (right)

CMR images were analysed offline by blinded readers at a core lab (Montreal Heart Institute, Montreal Canada) using certified software (cvi42, version 4, Circle Cardiovascular Imaging Inc., Calgary, Alberta, Canada). The cine SAx stack was used to perform quantitative LV/RV functional and volumetric evaluations. Epicardial and endocardial contours were traced manually or semi-automatically using the built-in “threshold tool” with attention to anatomy and potential image artefacts (Fig. 1). Anatomical accuracy was verified by carefully tracing contours for LV mass at end-systole, including papillary muscles and trabecular tissue into LV mass. There are several reasons for using the systolic phase for quantifying LV mass, as discussed in detail by Riffel et al. [15] Firstly, the overall length of the line defining the endocardial border is shorter and thus, a shorter length susceptible to errors. Secondly, the systolic phase typically has fewer slices than the diastolic phase, thus less contours to draw and less chances for errors. Finally, during systole, the inter-trabecular recesses are closed and therefore, there is lower probability for partial volume errors (Fig. 2). For additional reference, LV mass in end-diastole was also measured and reported in the supplementary materials.

For LV volume calculations, the entire outflow track was included into the end-diastolic and end-systolic phases. Consistent with the LV evaluation, RV volume contours were segmented, excluding the trabeculae and papillary muscles from the blood pool. Expert consensus from the research committee deemed RV mass measurements were not reliable to be used routinely in the clinical setting. Therefore, RV mass was not measured nor reported, so as to avoid confusion or misguidance.

Left atrial (LA) volume was derived from the long axis 2-chamber and 4-chamber views, according to the Biplane method [17]. Maximum LA volume was measured at end-systole, immediately before mitral valve opening and minimum LA volume was measured at end-diastole, immediately before mitral valve closure [17]. Image quality was qualitatively assessed by expert observers as either good, acceptable, or poor with consideration of anatomical structures or artefacts related to breathing, inadequate ECG triggering (blurring), flow, chemical shift (T2* susceptibility), or ghosting (T1 effects). Only participants with good or acceptable image quality were included in the final data analysis.

### Inter-observer and intra-observer quality assurance

Based on derived sample size calculations for intraclass correlation (ICC) [18], roughly 27 subjects (assuming there are 2 raters, 50 measurements in total) and 17 subjects (assuming there are 4 raters; 100 measurements in total) were estimated to detect ICC = 0.86 with 80% power. Therefore, the evaluation of intra- and inter-observer reliability was performed using 25 randomly selected CMR studies on four variables: end-diastolic volume (EDV), end-systolic volume (ESV), stroke volume (SV), and ejection fraction (EF) for both the LV and RV. CMR images with altered protocols, phantoms scans for quality control purposes, files sent more than once by the users, images sent with incorrect registry information, images not analyzable due to poor quality, and missing or incomplete CMR exams were excluded from this analysis. For the intra-observer reliability, the CMR studies were read twice by the assigned observers. A minimum delay of 4 weeks between the first and second readings was used to minimize observer bias. For inter-observer reliability, two readers were included in the analyses. The level of experience among the two observers ranged from expert (more than 15 + years of experience in CMR contouring and analysis) to 5 years of CMR contouring experience. The readers underwent standardized protocol training using practice cases to ensure contours were drawn accurately and consistently prior to analyzing participant data. As each reader made repeated measurements, methods described in Shrout and Fleiss [19] were not applicable. Hence, ICC were calculated to assess the inter- and intra-observer variability, as described in Eilasziw et al. [20] and visually depicted using Bland–Altman plots.

### Statistical analysis

Descriptive statistics for males and females were calculated for the risk factors and baseline characteristics and presented as mean (SD) or proportions (%). For continuous normal and non-normal variables, Two-sample T-test or Mann-Whitney U tests were used respectively to compare mean between females and men. Similarly, a Chi-square test was applied to compare the proportions. Means of CMR measurements between males and females were compared with two sample tests using a Satterthwaite approximation for the degrees of freedom. Normal reference ranges are defined as the 95% prediction interval, which is calculated by [12]

$$mean \, \pm \,t_{0.975, n - 1} \left( {SD} \right)\sqrt {{\raise0.7ex\hbox{${\left( {n + 1} \right)}$} \!\mathord{\left/ {\vphantom {{\left( {n + 1} \right)} n}}\right.\kern-\nulldelimiterspace} \!\lower0.7ex\hbox{$n$}} } .$$

For measured CMR variables (volume, mass) and derived variables (ejection fractions), reference ranges were calculated after excluding the outliers and presented in all the reference tables. All statistical analyses were performed using SAS (version 9.4, SAS Institute Inc., Cary, North Carolina, USA) and all figures were created using R (version 3.5.3. R Foundation for Statistical Computing, Vienna, Austria).

## Results

### Patient characteristics

A total of 8580 participants consented for the study with CMR data available for 8258 subjects. Due to history of CVD (10%) or missing LV parameters/poor image quality (5%), 1223 subjects were excluded (Additional file 1: Figure S1). Of the remaining 7035 participants, 3812 (54%) with history of CVD risk factors were excluded from the final analysis. Therefore, a total of 3,206 participants with CMR examinations were included in the analysis (2080 females, 65%). The mean age for males was 55.1 ± 8.8 and 55.2 ± 8.4 for females (p = 0.57). Details of the baseline demographics for the participants with CVD or CVD risk factors, by sex and age groups are shown in Table 1.

**Table 1:** Baseline demographics by sex and age groups for healthy participants (n = 3206)

Baseline variables	Age < 55 years		55 < = Age < 65 years		Age ≥ 65 years		Overall		p-value
	Female	Male	Female	Male	Female	Male	Female	Male	
	n = 972	n = 549	n = 795	n = 383	n = 313	n = 194	n = 2080	n = 1126	
Age, years	47.9 (4.7)	47.6 (4.8)	59.1 (2.8)	59.1 (2.9)	68.2 (2.5)	68.2 (2.6)	55.2 (8.4)	55.1 (8.8)	0.57
Body weight (kg)	64.0 (9.2)	79.5 (9.8)	62.9 (9.2)	77.7 (10.0)	62.3 (9.8)	75.3 (10.0)	63.3 (9.3)	78.2 (10.0)	< 0.001
Height (cm)	164.3 (6.2)	177.8 (7.0)	162.6 (6.5)	175.7 (7.0)	161.5 (6.9)	174.9 (6.2)	163.2 (6.5)	176.6 (7.0)	< 0.001
BMI	23.7 (2.9)	25.1 (2.5)	23.7 (2.9)	25.1 (2.4)	23.8 (3.0)	24.6 (2.7)	23.7 (2.9)	25.0 (2.5)	< 0.001
BMI > 30	0 (0.0%)	0 (0.0%)	0 (0.0%)	0 (0.0%)	0 (0.0%)	0 (0.0%)	0 (0.0%)	0 (0.0%)	
Percent body fat	30.9 (6.5)	20.7 (5.3)	31.9 (6.6)	21.5 (5.3)	32.7 (7.2)	22.0 (5.7)	31.5 (6.7)	21.2 (5.4)	< 0.001
Waist circumference (cm)	77.0 (8.7)	87.0 (8.4)	78.1 (9.0)	88.8 (8.4)	78.5 (9.3)	89.1 (9.0)	77.6 (8.9)	87.9 (8.5)	< 0.001
hip circumference (cm)	96.3 (8.5)	98.2 (6.9)	96.0 (8.2)	97.8 (5.9)	96.5 (8.2)	98.0 (6.3)	96.2 (8.4)	98.0 (6.5)	< 0.001
Systolic blood pressure	114.2 (10.9)	122.2 (9.6)	117.9 (11.4)	124.4 (9.6)	121.1 (11.0)	125.5 (9.6)	116.6 (11.4)	123.5 (9.7)	< 0.001
Diastolic blood pressure	73.9 (7.6)	77.1 (7.1)	74.2 (7.8)	76.6 (7.4)	73.0 (8.0)	76.3 (7.3)	73.9 (7.8)	76.8 (7.2)	< 0.001
Heart rate	69.7 (9.7)	67.0 (10.2)	69.7 (9.6)	65.6 (10.1)	70.8 (10.0)	67.8 (11.0)	69.9 (9.7)	66.7 (10.3)	< 0.001
Family history of cardiac disease	184 (18.9%)	73 (13.3%)	195 (24.5%)	83 (21.7%)	79 (25.2%)	40 (20.6%)	458 (22.0%)	196 (17.4%)	0.00
Ethnic background									
White Caucasian	733 (75.4%)	403 (73.4%)	610 (76.7%)	296 (77.3%)	261 (83.4%)	162 (83.5%)	1604 (77.1%)	861 (76.5%)	0.15
South Asian	43 (4.4%)	31 (5.6%)	23 (2.9%)	18 (4.7%)	4 (1.3%)	4 (2.1%)	70 (3.4%)	53 (4.7%)	
Chinese	171 (17.6%)	103 (18.8%)	142 (17.9%)	64 (16.7%)	43 (13.7%)	26 (13.4%)	356 (17.1%)	193 (17.1%)	
Other	25 (2.6%)	12 (2.2%)	20 (2.5%)	5 (1.3%)	5 (1.6%)	2 (1.0%)	50 (2.4%)	19 (1.7%)	
Medications									
Aspirin	15 (1.5%)	15 (2.7%)	29 (3.6%)	30 (7.8%)	19 (6.1%)	18 (9.3%)	63 (3.0%)	63 (5.6%)	< 0.001
Statin	0 (0.0%)	0 (0.0%)	0 (0.0%)	0 (0.0%)	0 (0.0%)	0 (0.0%)	0 (0.0%)	0 (0.0%)	

Values reported are mean (SD)

*BMI* body mass index

Overall, females had a lower BMI than males (p < 0.001), but a higher percentage of body fat (p < 0.001). Additionally, a small proportion in males (17%) and females (22%) reported a positive family history of CVD. Most subjects were white Caucasian (77% females, 77% men, p = 0.20). Approximately 17% were of Chinese ethnicity and approximately 4% were of South Asian ethnicity.

### Influence of sex on CMR-derived volumetric parameters

Table 2 shows sex-specific means ± SD for LV, RV and LA parameters indexed to body surface area (BSA) with derived normal reference ranges for all participants. The corresponding absolute values, and values indexed to height are provided in the Supplementary Materials (Additional file 2: Tables S1a–S1b). Absolute values, and values indexed to height or BSA for the different race/ethnicities are also reported for white Caucasians (Additional file 2: Table S4a–S4c), Chinese (Additional file 2: Table S5a–S5c), and South Asians populations (Additional file 2: Table S6a–S6c).

**Table 2:** Biventricular and left atrial reference values for healthy males (n = 1126) and females (n = 2080) indexed to body surface area (BSA)

CMR variables	Male				Female			
	Mean ± SD	95% CI*		Normal range	Mean ± SD	95% CI*		Normal range
		Lower limit	Upper limit			Lower limit	Upper limit	
LVEF (%)	62 ± 6	62	62	50–73	64 ± 6	64	65	53–76
LVSV, indexed to BSA (ml/m^2^)	46 ± 8	45	46	29–62	42 ± 7	42	42	28–56
LVEDV, indexed to BSA (ml/m^2^)	74 ± 13	73	75	48–100	65 ± 11	65	66	45–86
LVESV, indexed to BSA (ml/m^2^)	28 ± 7	28	29	14–43	23 ± 6	23	24	12–35
LV mass, indexed to BSA (g/m^2^)	61 ± 10	60	61	42–80	48 ± 8	47	48	33–62
LV mass to volume ratio (g/ml)	0.84 ± 0.16	0.83	0.848	0.53–1.15	0.74 ± 0.13	0.732	0.743	0.48–1.00
RVEF (%)	53 ± 6	53	53	41–65	58 ± 6	58	58	46–70
RVSV, indexed to BSA (ml/m^2^)	45 ± 8	45	46	29–62	42 ± 7	41	42	28–56
RVEDV, indexed to BSA (ml/m^2^)	86 ± 16	85	87	54–119	72 ± 13	72	73	47–8
RVESV, indexed to BSA (ml/m^2^)	41 ± 11	40	42	20–62	31 ± 8	30	31	14–47
Min. LA volume, indexed to BSA (ml/m^2^)	21 ± 7	21	21	7–35	19 ± 6	19	19	7–31
Max. LA volume, indexed to BSA (ml/m^2^)	39 ± 10	38	39	18–59	37 ± 9	36	37	19–54
LA EF (%)	46 ± 10	46	47	26–66	49 ± 11	48	49	28–70
LA SV, indexed to BSA (ml/m^2^)	18 ± 6	17	18	6–30	18 ± 6	18	18	7–29

*LV* Left ventricular; *RV* right ventricular; *EF* ejection fraction; *SV* stroke volume; *EDV* end-diastolic volume; *ESV* end-systolic volume; *LA* left atrium; *Min* minimum; *Max* maximum. Normal reference ranges are defined as the 95% prediction interval

Study cohort—excluded subjects with history of CVD or with risk factors of CVD -hypertension, diabetes, obesity, smoking or dyslipidemia

History of CVD—Aortic stenosis, Atrial fibrillation, Heart failure, Mitral stenosis, Previous PCI, Previous CABG, Valve surgery, TAVI, Hx of myocardial infarction

Reference ranges were calculated based on the formulae mean ± t_0.975,n−1_*sqrt[(n + 1)/n]*SD

*CI calculated based on the SE

**p value for testing Male vs Female

Overall, ventricular volumes and mass were greater in males than in females. The indexed mean LVSV (46 ml/m^2^ ± 8), LVEDV (74 ml/m^2^ ± 13), LVESV (28 ml/m^2^ ± 7), and LV mass (61 g/m^2^ ± 10) for males were significantly larger than the indexed LVSV (42 ml/m^2^ ± 7), LVEDV (65 ml/m^2^ ± 11), LVESV (23 ml/m^2^ ± 6), and LV mass (48 g/m^2^ ± 8) for females, respectively (all p < 0.001). Similarly, indexed RVSV (45 ml/m^2^ ± 8), RVEDV (86 ml/m^2^ ± 16) and RVESV (41 ml/m^2^ ± 11) for males were larger than indexed RVSV (42 ml/m^2^ ± 7), RVEDV (72 ml/m^2^ ± 13) and RVESV (31 ml/m^2^ ± 8) for females (all p < 0.001). However, LVEF (62% ± 6) and RVEF (53% ± 6) for males were significantly less than LVEF (64% ± 6) and RVEF (58% ± 6), (p < 0.001) for females.

### Age-STRATIFIED CMR-derived volumetric parameters

Age-stratified indexed reference ranges are shown for males and females in Tables 3 and 4, respectively. Corresponding absolute values (Additional file 2: Tables 2a and 2b) and values indexed to height (Additional file 2: Tables 3a and 3b) can be found in Supplementary Materials for males and females, respectively. Age groups were stratified into 4 clinically relevant categories by decade of age (35 ≤ to < 45 years, 45 ≤ to < 55 years, 55 ≤ to < 65 years, and 65 ≤ to < 75 years), with the greatest representation from the middle-aged population from 45 to 64 years (71% of males and 74% of females).

**Table 3:** Biventricular and left atrial reference values indexed to BSA for males age 35 to 75 years, stratified by 10-year age categories

CMR variables	35 ≤ Age < 45 years (n = 141)	45 ≤ Age < 55 years (n = 408)	55 < = Age < 65 years (n = 383)	65 ≤ Age < 75 years (n = 194)
LVEF, %	61 ± 6 (49–73)	62 ± 6 (50–73)	62 ± 6 (51–73)	62 ± 6 (50–75)
LVSV, indexed to BSA (ml/m^2^)	47 ± 8 (30–64)	46 ± 8 (30–62)	46 ± 8 (30–62)	43 ± 8 (26–59)
LVEDV, indexed to BSA (ml/m^2^)	77 ± 13 (50–103)	75 ± 13 (50–100)	74 ± 13 (48–100)	69 ± 13 (44–94)
LVESV, indexed to BSA (ml/m^2^)	30 ± 8 (15–46)	29 ± 7 (15–43)	28 ± 7 (14–43)	26 ± 7 (12–40)
LV mass, indexed to BSA (g/m^2^)	61 ± 11 (40–82)	61 ± 10 (42–80)	62 ± 10 (43–81)	58 ± 9 (40–75)
LV mass to volume ratio (g/ml)	0.81 ± 0.13 (0.54–1.07)	0.83 ± 0.15 (0.53–1.13)	0.85 ± 0.15 (0.56–1.14)	0.86 ± 0.20 (0.47–1.26)
RVEF, %	51 ± 6 (39–64)	53 ± 6 (41–64)	53 ± 6 (41–66)	54 ± 6 (41–66)
RVSV, indexed to BSA (ml/m^2^)	46 ± 9 (29–64)	46 ± 8 (30–62)	46 ± 8 (29–62)	43 ± 8 (26–59)
RVEDV, indexed to BSA (ml/m^2^)	91 ± 18 (56–126)	88 ± 16 (57–118)	86 ± 17 (54–119)	80 ± 15 (50–110)
RVESV, indexed to BSA (ml/m^2^)	45 ± 12 (21–69)	42 ± 10 (22–61)	41 ± 11 (19–63)	37 ± 10 (18–57)
Min. LA volume, indexed to BSA (ml/m^2^)	19 ± 6 (8–30)	20 ± 6 (8–33)	22 ± 8 (7–37)	21 ± 8 (5–37)
Max. LA volume, indexed to BSA (ml/m^2^)	36 ± 9 (17–54)	38 ± 10 (18–58)	40 ± 10 (20–61)	38 ± 11 (15–60)
LA SV, indexed to BSA (ml/m^2^)	46 ± 10 (27–65)	47 ± 10 (26–67)	46 ± 10 (27–65)	45 ± 11 (22–67)
LA EF (%)	17 ± 6 (5–28)	18 ± 6 (6–30)	19 ± 6 (7–31)	17 ± 6 (5–28)

Values are for males (n = 1126; age 35 to 75 years) reported as mean ± SD (normal range), stratified by 10-year categories. Indexed values are normalized to BSA. Normal reference ranges are defined as the 95% prediction interval (see Methods section for calculation)

Study cohort—excluded subjects with history of CVD or with risk factors of CVD -hypertension, diabetes, obesity, smoking or dyslipidemia

History of CVD—Aortic stenosis, Atrial fibrillation, Heart failure, Mitral stenosis, Previous PCI, Previous CABG, Valve surgery, TAVI, Hx of myocardial infarction

Reference ranges were calculated based on the formulae mean ± t_0.975,n−1_*sqrt[(n + 1)/n]*SD

*LV* Left ventricular; *RV* right ventricular; *EF* ejection fraction; *SV* stroke volume; *EDV* end-diastolic volume; *ESV* end-systolic volume; *LA* left atrium; *Min* minimum; *Max* maximum

*p value for testing if all the means are equal

**Table 4:** Ventricular and atrial reference values indexed to BSA for females age 35 to 75 years, stratified by 10-year age categories

CMR variables	35 ≤ Age < 45 years (n = 228)	45 ≤ Age < 55 years (n = 744)	55 < = Age < 65 years (n = 795)	65 ≤ Age < 75 years (n = 313)
LVEF, %	64 ± 5 (53–74)	64 ± 5 (54–75)	64 ± 6 (53–76)	65 ± 6 (54–77)
LVSV, indexed to BSA (ml/m^2^)	45 ± 7 (30–59)	43 ± 7 (29–57)	41 ± 7(28–55)	40 ± 6 (28–52)
LVEDV, indexed to BSA (ml/m^2^)	70 ± 11 (49–91)	67 ± 10 (47–88)	64 ± 10 (44–84)	62 ± 9 (44–79)
LVESV, indexed to BSA (ml/m^2^)	25 ± 6 (14–37)	24 ± 6 (13–35)	23 ± 6 (11–34)	21 ± 5 (11–32)
LV mass, indexed to BSA (g/m^2^)	48 ± 7 (33–62)	47 ± 8 (32–63)	48 ± 7 (33–63)	46 ± 8 (31–62)
LV mass to volume ratio (g/ml)	0.69 ± 0.11 (0.47–0.91)	0.72 ± 0.12 (0.47–0.96)	0.76 ± 0.14 (0.50–1.03)	0.76 ± 0.14 (0.49–1.04)
RVEF, %	56 ± 6 (45–68)	58 ± 6 (46–69)	58 ± 7 (45–71)	59 ± 6 (47–72)
RVESV, indexed to BSA (ml/m^2^)	44 ± 7 (30–59)	43 ± 7 (28–57)	41 ± 7 (27–54)	39 ± 6 (27–52)
RVEDV, indexed to BSA (ml/m^2^)	79 ± 13 (53–105)	74 ± 13 (49–100)	71 ± 13 (46–96)	67 ± 11 (46–88)
RVESV, indexed to BSA (ml/m^2^)	35 ± 8 (18–52)	32 ± 8 (16–48)	30 ± 8 (13–47)	28 ± 7 (14–42)
Min. LA volume, indexed to BSA(ml/m^2^)	17 ± 5 ( 6–27)	18 ± 6 (7–29)	19 ± 6 (7–32)	21 ± 7 (7–34)
Max. LA volume, indexed to BSA(ml/m^2^)	34 ± 8 (19–50)	37 ± 9 (20–54)	37 ± 9 (19–55)	38 ± 9 (20–57)
LA SV, indexed to BSA(ml/m^2^)	52 ± 11 (30–73)	50 ± 10 (30–71)	48 ± 11 (26–69)	47 ± 10 (27–67)
LA EF (%)	18 ± 5 ( 7–28)	18 ± 6 (7–30)	18 ± 6 (6–29)	18 ± 6 (7–29)

Values are for females (n = 2080, age 35 to 75 years), reported as mean ± SD (normal range), stratified by 10-year categories. Indexed values are normalized to BSA. Normal reference ranges are defined as the 95% prediction interval

Study cohort—excluded subjects with history of CVD or with risk factors of CVD -hypertension, diabetes, obesity, smoking or dyslipidemia

History of CVD—Aortic stenosis, Atrial fibrillation, Heart failure, Mitral stenosis, Previous PCI, Previous CABG, Valve surgery, TAVI, Hx of myocardial infarction

Reference ranges were calculated based on the formulae mean ± t_0.975,n−1_*sqrt[(n + 1)/n]*SD

*LV* Left ventricular; *RV* right ventricular; *EF* ejection fraction; *SV* stroke volume; *EDV* end-diastolic volume; *ESV* end-systolic volume; *LA* left atrium; *Min* minimum; *Max* maximum

*p value for testing if all the means are equal

Overall, indexed LVESV and LVEDV decreased as age increased (Additional file 1: Figure S2A and S2B) for both males and females (p < 0.001). Indexed LV mass also decreased with advancing age for males (p < 0.001) and females (p < 0.001) (not shown). The same was seen with indexed RV end-diastolic and end-systolic volumes for males and females (not shown, p < 0.001). For men, there was no significant difference between LVEF across the different age strata (Additional file 1: Fig. 2C, p = 0.1985), but RVEF did increase with advancing age (not shown, p = 0.009). For females, there were significant differences seen in the LVEF and RVEF, whereby the mean EF values increased with advancing age (Fig. 2C, p < 0.001).

### Intra- and inter-observer Reliability

A summary of the inter and intra-observer reliability are listed in Table 5. Moderate to excellent intra- and inter-observer variability were demonstrated for all measured parameters. Representative examples of Bland–Altman plots for LV stroke volume are shown in Additional file 1: Figures S3 and S4.

**Table 5:** Intraclass correlation coefficient for inter-observer and intra-observer variability of CMR data

	Intra-observer reliability readers random^a^	Inter-observer reliability readers random^a^
LVEDV	0.88	0.92
LVESV	0.83	0.85
LVSV	0.85	0.87
LVEF	0.72	0.61
RVEDV	0.87	0.90
RVESV	0.90	0.90
RVSV	0.81	0.86
RVEF	0.86	0.83

^a^Readers are a representative of a large population of readers

*LV* Left ventricular; *RV* right ventricular; *EF* ejection fraction; *SV* stroke volume; *EDV* end-diastolic volume; *ESV* end-systolic volume

## Discussion

Among participants without known CVD or CV risk factors included in this large multi-ethnic population-based sample of 3206 adults aged 35–75 years, we provide accurate age- and sex-specific CMR-derived reference values for biventricular volumes, function, and mass and LA volume. Parameters normalized to BSA and height, as well as absolute values were reported, as volumetric parameters and mass are correlated with body habitus [5, 21]. BSA, which accounts for both height and weight, is the adjustment standard recommended by other societies, including those for echocardiography. [22] Clinical-decision making based solely on absolute values, while convenient, allow for potential under- or overestimation of chamber volumes and mass, undermining the utility of CMR.

### Strength and novelty of the study cohort

Multiple areas highlight the strength and novelty of the CAHHM CMR cohort, including the large recruitment of participants from a multi-ethnic population, absence of confounding pathological cardiovascular conditions, good representation of females, and finally, adherence to high imaging standards for quality assurance (Table 6). Previous studies have sought to provide CMR-based reference values for the clinical assessment of ventricular and atrial parameters. However, our sample population is the largest known to date, with prior sample sizes ranging from 60 participants [9] to the most recent publication by Petersen et al. that included 800 subjects for analysis [12] (Table 6). A large sample population ensures more accurate mean reference values, identification of outliers, and overall smaller margins of errors. This is particularly important in conditions such as heart failure with preserved ejection fraction that require highly accurate evaluation of ventricular function and volume to properly guide medical therapy, as research now demonstrate increased mortality when LVEF exceeds 65% [23, 24].

**Table 6:** Comparison between studies with CMR-derived normal reference values

Study (Author, Journal Year)	Sample Size (n =)	Age (years)	Ethnicity	% Female representation (male:female)	Contouring Method	Mean LVEDV indexed to BSA (ml/m^2^)	Mean LVEF (%)
Current study, Luu et al. 2021	3206	35–75	Multi-ethnic	65% (1126:2080)	Inclusion of papillary muscle in LV mass	Female: 66 Male: 74	Female: 65 Male: 62
Petersen et al. JCMR 2017	800	45–74	All Caucasians	54% (368:432)	Inclusion of papillary muscle in LV volume	Female: 74 Male: 85	Female: 61 Male: 58
Lei et al., JMRI 2017	120	23–83	All Han Chinese	50% (60:60)	Inclusion of papillary muscle in LV volume	Female: 68.7 Male: 76.5	Female: 67.1 Male: 64.6
Le et al., JCMR 2016	180	20–69	All Singaporean Chinese	49% (91:89)	Inclusion of papillary muscle in LV mass	Female: 71 Male: 79	Female: 62 Male: 58
Yeon et al., JMRI 2015	852	38–88^a^	Most Caucasians	60% (512:340)	Inclusion of papillary muscle in LV volume	Female: 62 Male: 71	Female: 60.6 Male: 58.6
Natori et al., AJR 2006	800	45–84	Multi-ethnic	50% (400:400)	Inclusion of papillary muscle in LV volume	Female: 64.5 Male: 73.9	Female:71.8 Male: 67.2
Maceira et al., JCMR 2006	120	20–80	Not mentioned	50% (60:60)	Inclusion of papillary muscle in LV mass	Female: 75 Male: 80	Female: 67 Male: 67
Hudsmith et al., JCMR 2005	108	21–68	Not mentioned	42% (63:45)	Inclusion of papillary muscle in LV mass	Female: 78 Male: 82	Female: 69 Male: 69
Alfakih et al., JMRI 2003	60	20–65	Not mentioned	50% (30:30)	Inclusion of papillary muscle in LV mass	Female: 77.7 Male: 82.3	Female: 64.0 Male: 64.2
Salton et al., JACC 2002	142	38–72	Not mentioned	56% (63:79)	Inclusion of papillary muscle in LV volume	Female: 49.8 Male: 57.6	Female: 70 Male: 69

^a^Age range not reported in original publication, but study population clarified in the source by Oyama et al. 2008. “Differential Impact of Age, Sex, and Hypertension on Aortic Atherosclerosis.” *Arteriosclerosis, Thrombosis, and Vascular Biology* 28 (1): 155–59

*LV* left ventricular; *LVEDV* left ventricular end-diastolic volume; *BSA* body surface area; *LVEF* left ventricular ejection fraction

Despite growing momentum for the increasing utility of CMR in clinical routine, there is lack of agreement on specific standards for quantitative parameters. The latest Society for Cardiovascular Magnetic Resonance (SCMR) 2018 expert consensus document for imaging endpoints recognize that there is moderate variability in normal ranges depending on the population studied and method of quantification [1]. The recommended resource, however, for normal values as per the SCMR is from a review by Kawel-Boehm et al. that summarized the findings of three publications using the bSSFP CMR sequence of very small sample sizes and heterogeneous ethnic population by Alfakih et al. 2003 (n = 60), Hudsmith et al. (n = 108), and Maceira et al. (n = 120) (Table 6). [11] Thus, as expected with small sample sizes, there were differences between findings by Kawel-Boehm et al. and our own. For instance, the ventricular reference values for indexed LVEDV and LV mass reported by Kawel-Boehm et al. were different by nearly 10 ml/m^2^ when compared to the sex-specific values from our study, which incorporated data from over 3000 individuals. Furthermore, Kawel-Boehm et al. provide adult parameters for age stratified by younger or older than 60 years of age and not specific reference values by age decade, limiting the robust clinical application.

With strict adherence to the research protocols outlined by the CAHHM committee, substantial efforts were made to ensure the study population was indeed free of underlying CVD or risk factors, excluding over 64% of consented participants. The sample population also included participants from different race/ethnicities including white Caucasians, South Asians, and Chinese descent, allowing for increased generalizability and broad application of the reference values. Researchers have previously reported variability in LV volumes and mass across the different races/ethnicities and as such, we provide normal references values for the separate groups in the supplemental materials [25]. We also report smaller indexed LV volumes and mass in the Chinese population compared to subjects from European descent (white Caucasians) [14, 26], and note similar volumes and mass in the South Asians compared to the white Caucasians.

**Fig. 3:**
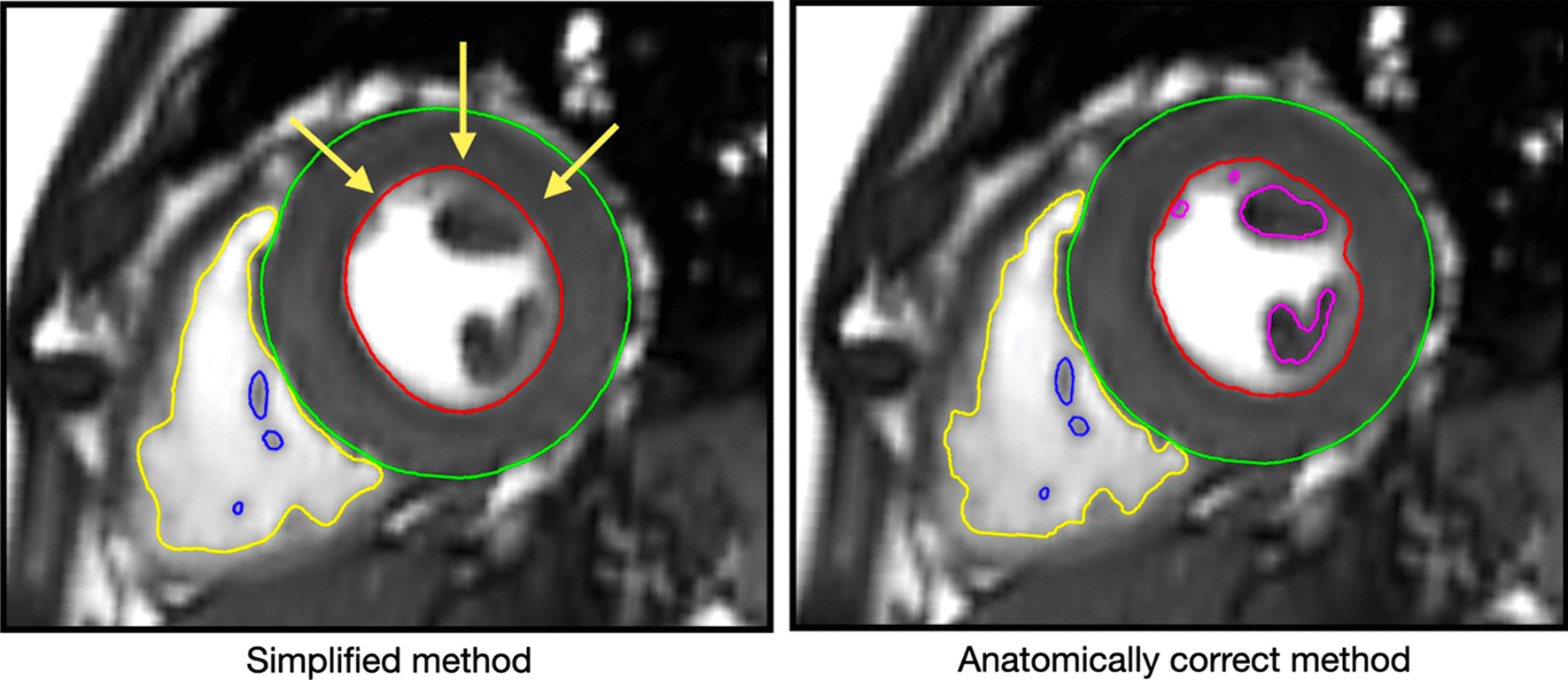
Anatomically correct contouring method for left ventricular (LV) mass. Papillary muscles and trabecular structures are included as myocardium (and not as blood) (right panel). Using the simplified method (left) of drawing the contour to define the arbitrary “cutoff” line (yellow arrows) may be subjected to additional observer variability and errors

When comparing our results to the previous reference values reported by Petersen et al. using the United Kingdom (UK) Biobank cohort, notable differences in the indexed mean LV and RV parameters were seen. For example, normal indexed LVEDV for females in our study was 65 ml/m^2^ ± 11 (range 45 to 86 ml/m^2^), which is different when compared to the normal indexed LVEDV by Petersen et al. for overall females of 74 ml/m^2^ ± 12 (range 54 to 94 ml/m^2^). We suspect the discrepancy may be due to the different contouring approach, which is an important contribution of our normative data to the current literature. In contrast to some previous studies, our contouring method of including the papillary muscles and trabecular structures as myocardial mass (and not as blood) is anatomically and functionally correct [15], and recommended by the recently updated recommendations of the SCMR [27]. The exclusion of papillary muscles from LV mass has been shown to lead to an underestimation of LV hypertrophy [28]. Opponents may argue that this contouring approach suffers from partial volume effects, averaging trabeculations with the blood pool, thus leading to overestimation of LV mass and underestimation of LV volume. However, the problem of partial volume may actually lead to errors in both directions, i.e. under- and overestimation [29]. Using the simplified method of drawing the contour to define the arbitrary “cutoff” line may be subjected to additional observer variability and errors (Fig. 3). We emphasize that several previous studies have also used the anatomically correct contouring [9, 13, 30], with ex vivo validations showing very good agreement [31, 32] and researchers demonstrated that this method provides more reliable values [15]. Yet, many centers and even large cohort studies, such as the UK Biobank [33] use a simplified method that cuts off papillary and trabecular tissue, explaining the larger volumes observed in the UK Biobank cohort. Recommendations concede that such a simplification is inaccurate, but “allow” for this simplification for practicality reasons [34]. Our study is the largest cohort to utilize the anatomically and functionally correct contouring method to date, demonstrating its feasibility for clinical and research settings.

### Dependence of values on sex and age on LV and RV volumes, function, and mass

It has long been known that sex has significant independent influence on normal values for biventricular volumes and mass [35]. Similarly, our study found that LV and RV volumes were significantly smaller in females compared to men, and that LV mass was also larger in males than females [35]. Age also has a significant influence on ventricular volumes, whereby LV and RV volumes are known to decrease with advancing age, with significant differences between each age strata [12, 21]. The influence of age on indexed LV mass to BSA, however, has not been well understood. Among the studies that specifically included age-specific reference ranges or age-associated statistical analyses—using similar CMR protocols with a cine bSSFP approach—the UK Biobank observed, upon normalization to BSA, LV mass did not change significantly with age in either sex [12]. Le Ven et al. in their study of 434 white Caucasian adults without CVD or risk factors, reported that, while age had an independent influence on most ventricular measurements, it was not significantly associated with LV mass [13]. In our study, the influence of age was significant on indexed LV mass in both sexes, likely due to the inclusion of papillary muscles, which reduces accuracy compared to autopsy, but results in higher precision, smaller observer variability, and allows for more robust clinical application [1].

We found values of LVEF and RVEF were significantly higher for females due to higher stroke volumes, which is consistent with previous large CMR studies, including the Dallas Heart Study and trials using computed tomography [2, 12, 36]. LVEF and RVEF increases with age in both sexes, with a more pronounced relationship seen in females than men. However, consistent with previous findings, the normal reference ranges across the different age strata remained similar [12].

### Study limitations

Firstly, while the overall composition of the study population included participants from different ethnicities, the majority were of white Caucasian background, which may limit the overall generalizability of the measurements. However, measurements were indexed to BSA to help reduce the confounding effects of ethnicity. Secondly, based on considerations for feasibility, cost, and research limitations, observer variability was performed in 25 cases (representing ~ 10% of the study population) for only RV and LV parameters, measurements which frequently show inconsistencies in the clinical environment. Our study demonstrated overall good quality assurance and based on sample size calculations, the addition of more cases or readers would not contribute further meaningful findings. Lastly, owing to logistical issues, normative values for RA data was not provided in this paper. An alternative paper will be released to separately address accurate measurements of RA and RV parameters. The primary focus for this paper, however, was to provide a robust set of normal reference values for ventricular parameters using the anatomically correct contouring method. Therefore, the results of this study still adds significant value to existing normative references.

## Conclusion

Recognizing the significant influence of sex and age on volumetric parameters is particularly important in the clinical evaluation of several cardiovascular conditions. Using anatomically correct contouring methodology, this large, multi-ethnic cohort from the Canadian Alliance for Healthy Heart and Minds offers a robust set of CMR-derived sex and age-specific reference values that can be used to distinguish cardiac impairment in clinical and research settings.

## Supplementary Information

**Additional file 1: Figure S1.** Flow chartfor patient selection. RI, magnetic resonance imaging; LVEF, leftventricular ejection fraction; LV mass, left ventricular mass; CVD,cardiovascular disease; PURE, prospective urban and rural epidemiologicalstudy; CPTP, the Canadian Partnership for Tomorrow Project; BC Generations,British Columbia; OHS, Ontario Health Study; Atlantic PATH, AtlanticPartnership for Tomorrow's Health; MHI, Montreal Heart Institute. **Figure S2**. Age-specific trends for males and females for A) LV end-systolic volumesindexed to BSA (ml/m^2^); B) LV end-diastolic volumes indexed to BSA(ml/m^2^); and C) LVEF (%). Linear regression was applied to model the data,which are presented as mean (blue lines) and 95% confidence intervals (redlines). **Figure S3**. Representativeexamples of Bland Altman plots for inter-observer variability of absolute leftand right ventricular stroke volumes (ml). **Figure S4.** Representativeexamples of Bland Altman plots for intra-observer variability of absolute leftand right ventricular stroke volumes (ml).

**Additional file 2: Table S1a.** Biventricular and left atrial absolute reference valuesfor healthy males (n=1126) and females (2080). Values reported as mean±SDwith 95% confidence intervals and normal ranges. **Table S1b.** Biventricular and left atrialreference values indexed to height for healthy males (n=1126)and females (2080). Values reported as mean±SD with 95% confidenceintervals and normal ranges. **Table S2a.** Biventricular and left atrialabsolute reference values for males 35 to 75 years, stratifiedby 10–year age categories. Values reported as mean±SDwith normal ranges. **Table S2b.** Biventricular and left atrial reference values indexedto height for males 35 to 75 years, stratified by 10–year agecategories. Values reported as mean±SD with normal ranges. **Table S3a.** Biventricular and left atrial absolute referencevalues for females 35 to 75 years, stratified by 10–yearage categories. Values reported as mean±SDwith normal ranges. **Table S3b.** Biventricular and left atrial reference values indexedto height for females 35 to 75 years, stratified by 10–yearage categories. Values reported as mean±SDwith normal ranges. **Table S4a.** Biventricular and atrial absolute reference values for healthy males (n=861) and females (1604) for white Caucasiansonly. Values reported as mean±SD with 95% confidence intervals and normal ranges. **Table S4b.** Biventricular and left atrial reference values index toheight for healthy males (n=861) and females (1604) for white Caucasiansonly. Values reported as mean±SD with 95% confidence intervals and normal ranges. **Table S4c.** Biventricular and atrial reference values indexed to BSAfor healthy males (n=861) and females (1604) for white Caucasiansonly. Values reported as mean±SD with 95% confidence intervals and normal ranges. **Table S5a.** Biventricular and left atrial absolute reference valuesfor healthy males (n=191) and females (356) for Chinese only.Values reported as mean±SD with 95% confidence intervals and normal ranges. **Table S5b.** Biventricular and left atrial reference values indexed toheight for healthy males (n=193) and females (356) for Chinese only.Values reported as mean±SD with 95% confidence intervals and normal ranges. **Table S5c** Biventricular and left atrial reference values indexed toBSA for healthy males (n=193) and females (356) for Chinese only.Values reported as mean±SD with 95% confidence intervals and normal ranges. **Table S6a.** Biventricular and left atrial absolute reference valuesfor healthy males (n=53) and females (70) for South Asians only.Values reported as mean±SD with 95% confidence intervals and normal ranges. **Table S6b.** Biventricular and atrial reference values indexed to heightfor healthy males (n=53) and females (70) for South Asians only.Values reported as mean±SD with 95% confidence intervals and normal ranges. **Table S6c.** Biventricular and atrial reference values indexed to BSAfor healthy males (n=53) and females (70) for South Asians only.Values reported as mean±SD with 95% confidence intervals and normal ranges. 

## Data Availability

The datasets generated during and/or analysed during the current study are available from the corresponding author on reasonable request.

## Abbreviations

BMI: Body mass index
BSA: Body surface area
bSSFP: Balanced steady state free precession
CAHHM: Conadian Alliance for Healthy Heart and Minds
CMR: Cardiovascular magnetic resonance
CVD: Cardiovascular disease
ECG: Electrocardiogram
EDV: End-diastolic volume
EF: Ejection fraction
ESV: End-systolic volume
LA: Left atrium/left atrial
LV: Left ventricle/left ventricular
MRI: Magnetic resonance imaging
RV: Right ventricle/right ventricular
SAx: Short axis
SCMR: Society for Cardiovascular Magnetic Resonance
SV: Stroke volume
