# Supplementary material for: Normal sex and age-specific parameters in a multi-ethnic population: a cardiovascular magnetic resonance study of the Canadian Alliance for Healthy Hearts and Minds cohort
Source: J Cardiovasc Magn Reson. 2022 Jan 3;24:2. doi: 10.1186/s12968-021-00819-z (PMC8722350; doi:10.1186/s12968-021-00819-z)

**Suppl Figure 1: Flow chart for patient selection.**

MRI, magnetic resonance imaging; LVEF, left ventricular ejection fraction; LV mass, left ventricular mass; CVD, cardiovascular disease; PURE, prospective urban and rural epidemiological study; CPTP, the Canadian Partnership for Tomorrow Project; BC Generations, British Columbia; OHS, Ontario Health Study; Atlantic PATH, Atlantic Partnership for Tomorrow's Health; MHI, Montreal Heart Institute.


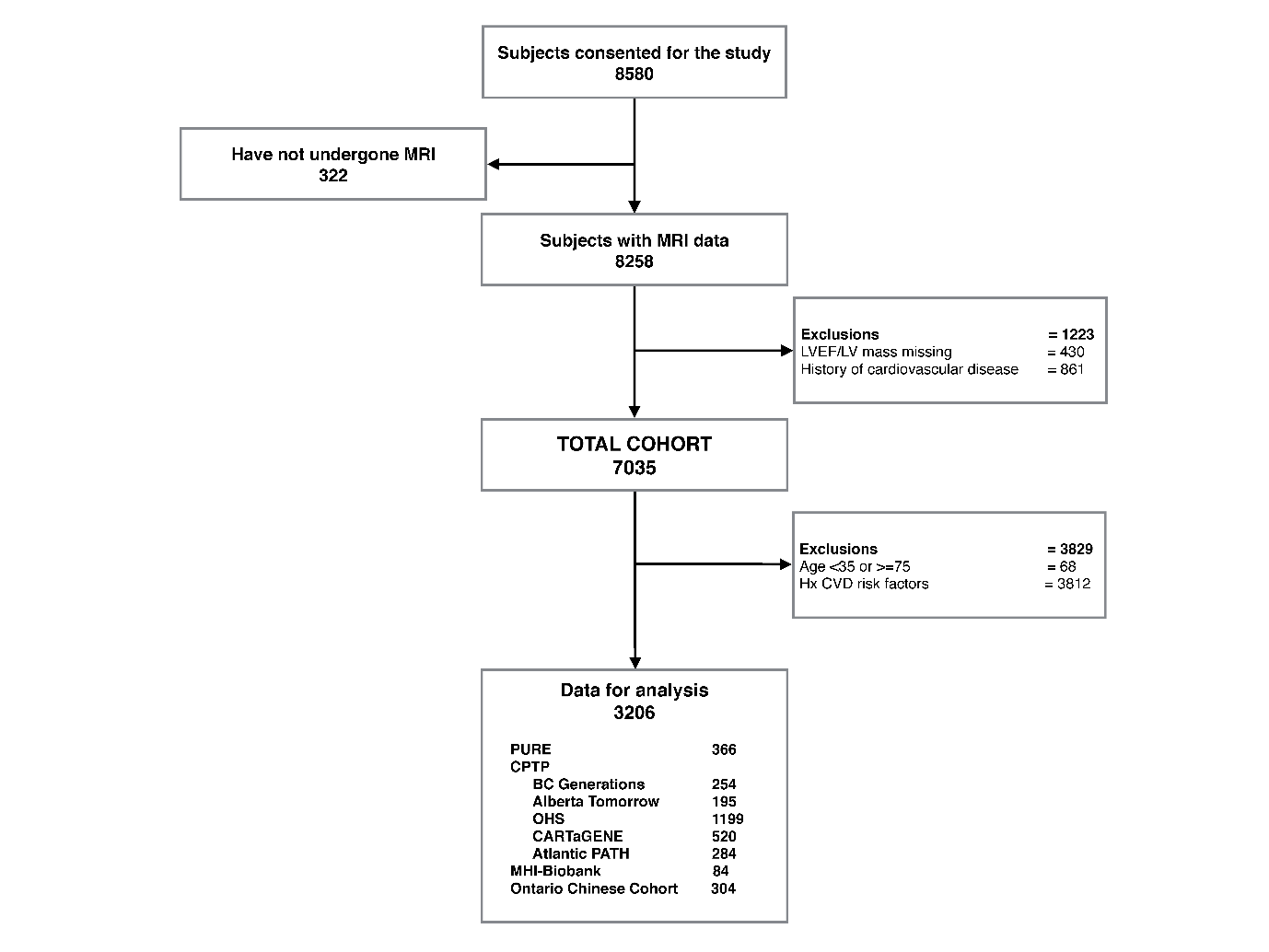


**Suppl Figure 2:** Age-specific trends for males and females for A) LV end-systolic volumes indexed to BSA (ml/m^2^); B) LV end-diastolic volumes indexed to BSA (ml/m^2^); and C) LVEF (%). Linear regression was applied to model the data, which are presented as mean (blue lines) and 95% confidence intervals (red lines).


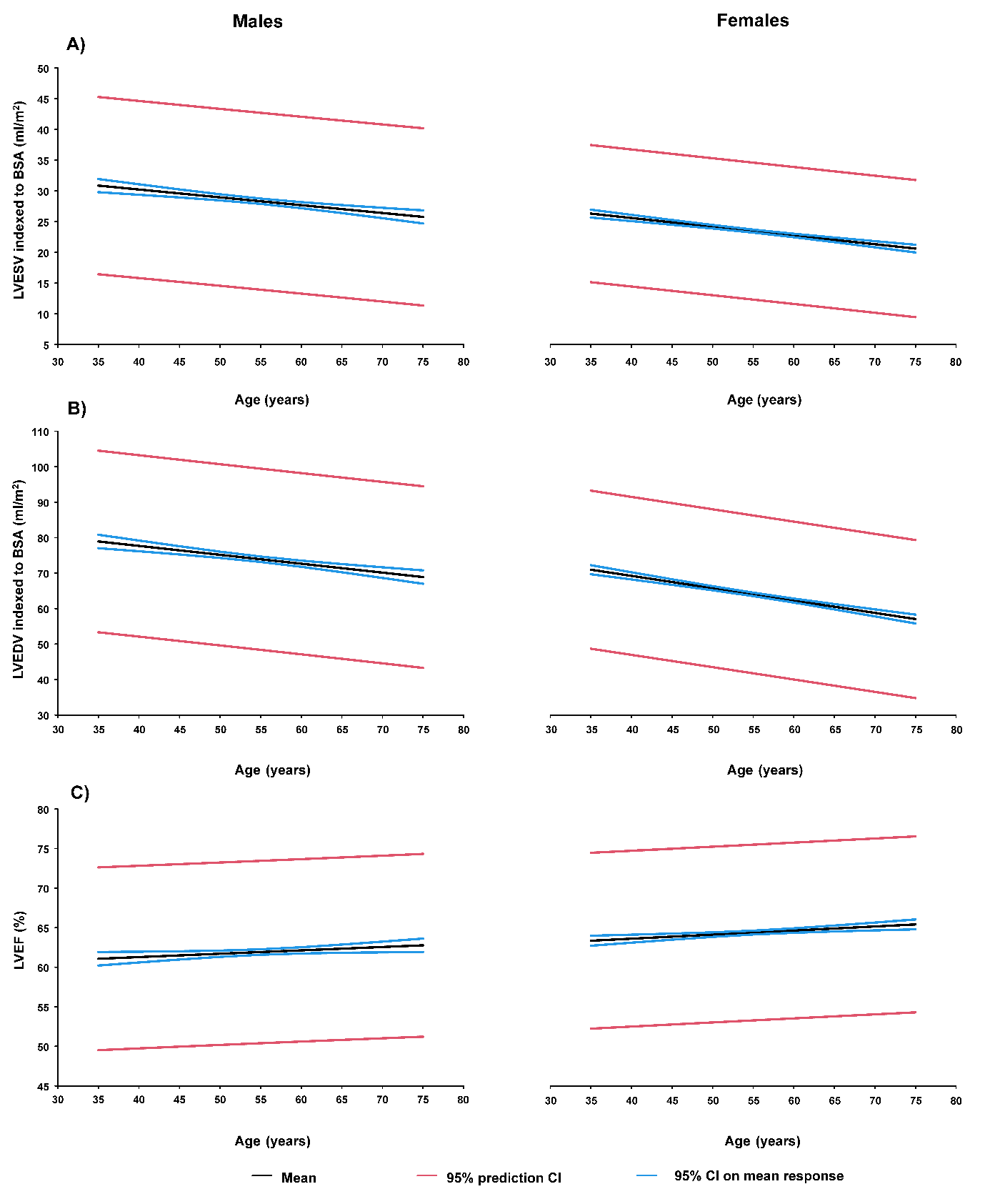


**Suppl Figure 3:** Representative examples of Bland Altman plots for inter-observer variability of absolute left and right ventricular stroke volumes (ml).


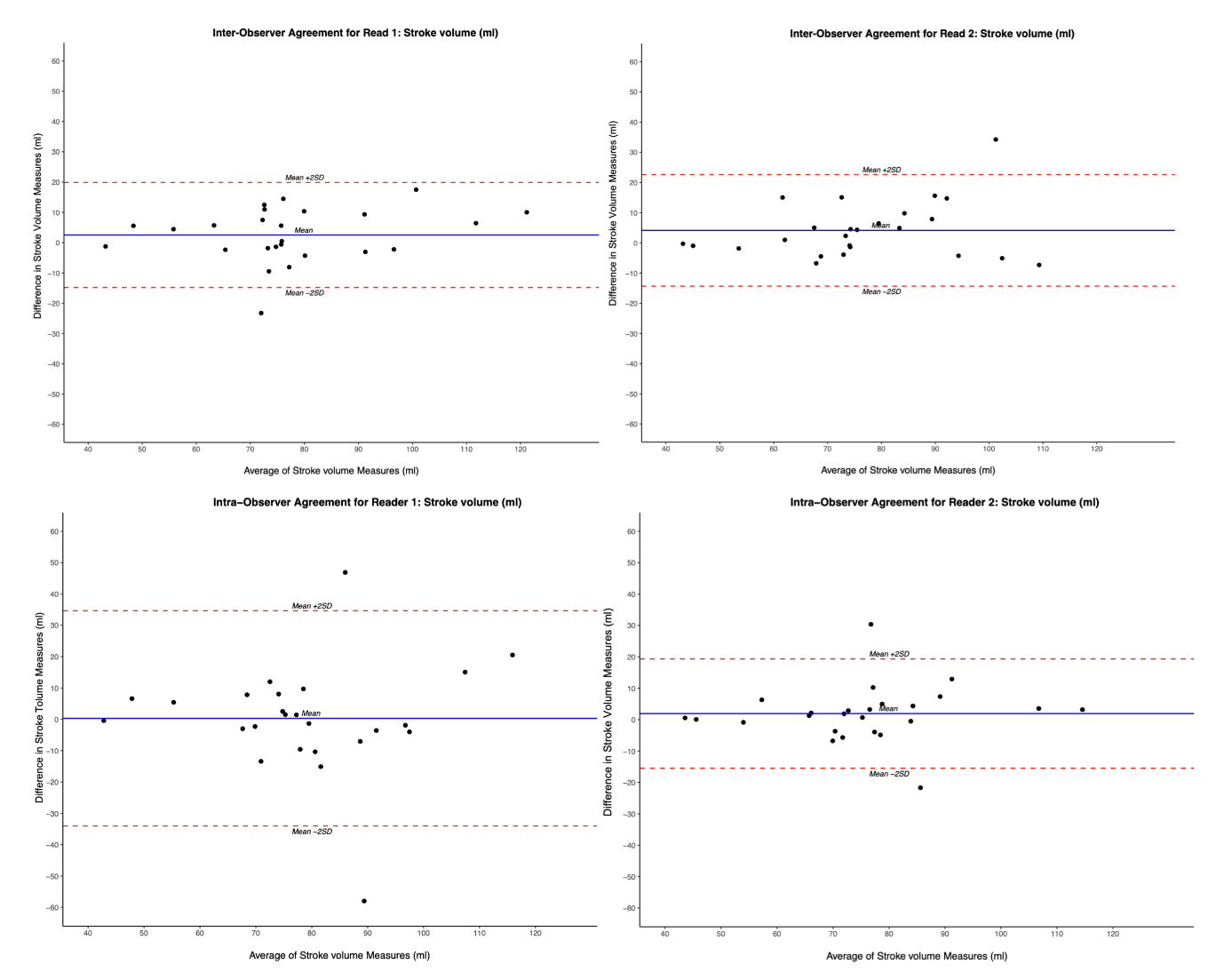


**Suppl Figure 4:** Representative examples of Bland Altman plots for intra-observer variability of absolute left and right ventricular stroke volumes (ml).


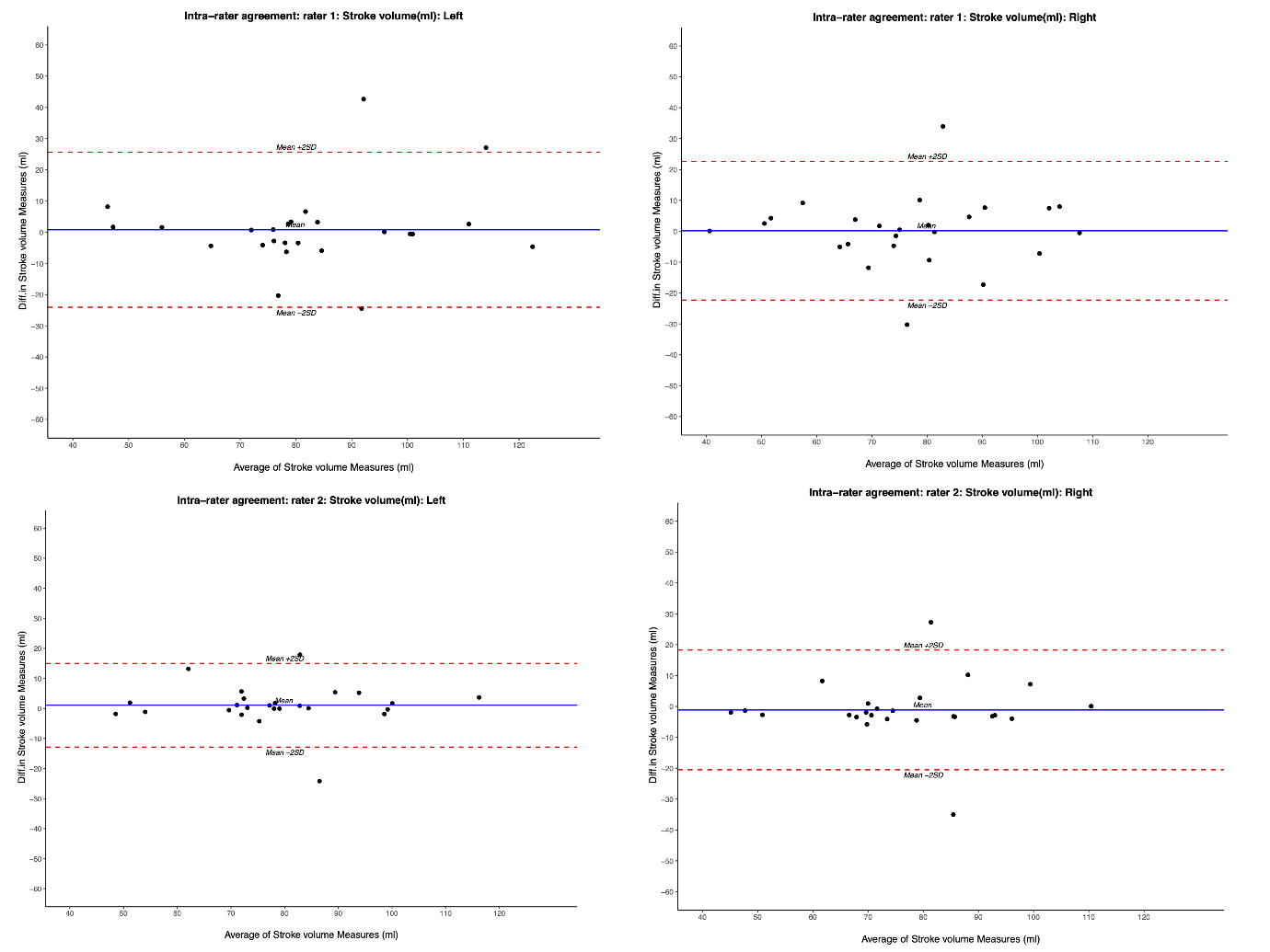

Supplement: Supplementary file 1 — Additional file 1: Figure S1. Flow chartfor patient selection. RI, magnetic resonance imaging; LVEF, leftventricular ejection fraction; LV mass, left ventricular mass; CVD,cardiovascular disease; PURE, prospective urban and rural epidemiologicalstudy; CPTP, the Canadian Partnership for Tomorrow Project; BC Generations,British Columbia; OHS, Ontario Health Study; Atlantic PATH, AtlanticPartnership for Tomorrow's Health; MHI, Montreal Heart Institute. Figure S2. Age-specific trends for males and females for A) LV end-systolic volumesindexed to BSA (ml/m2); B) LV end-diastolic volumes indexed to BSA(ml/m2); and C) LVEF (%). Linear regression was applied to model the data,which are presented as mean (blue lines) and 95% confidence intervals (redlines). Figure S3. Representativeexamples of Bland Altman plots for inter-observer variability of absolute leftand right ventricular stroke volumes (ml). Figure S4. Representativeexamples of Bland Altman plots for intra-observer variability of absolute leftand right ventricular stroke volumes (ml). [file 12968_2021_819_MOESM1_ESM.docx]
