# Supplementary material for: Normal sex and age-specific parameters in a multi-ethnic population: a cardiovascular magnetic resonance study of the Canadian Alliance for Healthy Hearts and Minds cohort
Source: J Cardiovasc Magn Reson. 2022 Jan 3;24:2. doi: 10.1186/s12968-021-00819-z (PMC8722350; doi:10.1186/s12968-021-00819-z)
Supplement: Supplementary file 2 — Additional file 2: Table S1a. Biventricular and left atrial absolute reference valuesfor healthy males (n=1126) and females (2080). Values reported as mean±SDwith 95% confidence intervals and normal ranges. Table S1b. Biventricular and left atrialreference values indexed to height for healthy males (n=1126)and females (2080). Values reported as mean±SD with 95% confidenceintervals and normal ranges. Table S2a. Biventricular and left atrialabsolute reference values for males 35 to 75 years, stratifiedby 10–year age categories. Values reported as mean±SDwith normal ranges. Table S2b. Biventricular and left atrial reference values indexedto height for males 35 to 75 years, stratified by 10–year agecategories. Values reported as mean±SD with normal ranges. Table S3a. Biventricular and left atrial absolute referencevalues for females 35 to 75 years, stratified by 10–yearage categories. Values reported as mean±SDwith normal ranges. Table S3b. Biventricular and left atrial reference values indexedto height for females 35 to 75 years, stratified by 10–yearage categories. Values reported as mean±SDwith normal ranges. Table S4a. Biventricular and atrial absolute reference values for healthy males (n=861) and females (1604) for white Caucasiansonly. Values reported as mean±SD with 95% confidence intervals and normal ranges. Table S4b. Biventricular and left atrial reference values index toheight for healthy males (n=861) and females (1604) for white Caucasiansonly. Values reported as mean±SD with 95% confidence intervals and normal ranges. Table S4c. Biventricular and atrial reference values indexed to BSAfor healthy males (n=861) and females (1604) for white Caucasiansonly. Values reported as mean±SD with 95% confidence intervals and normal ranges. Table S5a. Biventricular and left atrial absolute reference valuesfor healthy males (n=191) and females (356) for Chinese only.Values reported as mean±SD with 95% confidence intervals and normal ranges. Table S5b. Biventricular and [file 12968_2021_819_MOESM2_ESM.docx]

**Suppl Table 1a: Biventricular and left atrial absolute reference values for healthy males (n=1126) and females (2080). Values reported as mean±SD with 95% confidence intervals and normal ranges.**

|  | **Males** | | | | **Females** | | | |
| --- | --- | --- | --- | --- | --- | --- | --- | --- |
| **CMR variables** | **Mean ±SD** | **95% CI*** | | **Normal range** | **Mean ±SD** | **95% CI*** | | **Normal range** |
|  | | **Lower limit** | **Upper limit** |  |  | **Lower limit** | **Upper limit** |  |
| LV ejection fraction (%) | 62 ± 6 | 62 | 62 | 50- 73 | 64 ± 6 | 64 | 65 | 53- 76 |
| LV stroke volume (ml) | 89 ± 18 | 88 | 90 | 54-125 | 71 ± 14 | 71 | 72 | 44- 98 |
| LV end systolic volume (ml) | 56 ± 15 | 55 | 56 | 25- 86 | 40 ± 11 | 39 | 40 | 19- 61 |
| LV end diastolic volume (ml) | 145 ± 29 | 143 | 147 | 88-202 | 111 ± 21 | 110 | 112 | 70-152 |
| LV myocardial mass systole (g) | 119 ± 22 | 118 | 120 | 76-162 | 81 ± 15 | 80 | 81 | 51-110 |
| LV myocardial mass diastole (g) | 114 ± 21 | 113 | 115 | 72-156 | 78 ± 15 | 77 | 79 | 49-107 |
| LV mass to volume ratio (g/ml) | 0.84 ±0.16 | 0.83 | 0.85 | 0.53-1.15 | 0.74 ±0.13 | 0.73 | 0.74 | 0.48-1.00 |
|  |  |  |  |  |  |  |  |  |
| RV ejection fraction (%) | 53 ± 6 | 53 | 53 | 41- 65 | 58 ± 6 | 58 | 58 | 46- 70 |
| RV stroke volume (ml) | 89 ± 18 | 88 | 90 | 53-124 | 70 ± 14 | 70 | 71 | 44- 97 |
| RV end systolic volume (ml) | 80 ± 23 | 79 | 82 | 35-125 | 52 ± 15 | 52 | 53 | 23- 81 |
| RV end diastolic volume (ml) | 169 ± 36 | 167 | 171 | 99-240 | 123 ± 25 | 122 | 124 | 74-171 |
|  |  |  |  |  |  |  |  |  |
| LA minimum | 41 ± 14 | 40 | 42 | 13- 69 | 32 ± 11 | 31 | 32 | 10- 53 |
| LA maximum | 75 ± 21 | 74 | 77 | 34-117 | 62 ± 16 | 62 | 63 | 30- 95 |
| LA ejection fraction (%) | 46 ± 10 | 46 | 47 | 26- 66 | 49 ± 11 | 48 | 49 | 28- 70 |
| LA stroke volume (ml) | 35 ± 12 | 34 | 36 | 11- 58 | 31 ± 10 | 30 | 31 | 11- 50 |

***Study cohort – excluded subjects with history of cardiovascular disease (CVD) or with risk factors of CVD –hypertension, diabetes, obesity, smoking or dyslipidemia***

***History of CVD – Aortic stenosis, Atrial fibrillation, Heart failure, Mitral stenosis, Previous percutaneous coronary intervention (PCI), Previous coronary artery bypass graft surgery (CABG), Valve surgery, TAVI, Hx of myocardial infarction***

**** – CI calculated based on the SE***

***** – P value for testing males vs females***

***Reference ranges were calculated based on the formulae  mean ± t_0.975,n–1_*sqrt[(n+1)/n]*SD***

LV, left ventricular; RV, right ventricular; LA, left atrial.

**Suppl Table 1b: Biventricular and left atrial reference values indexed to height for healthy males (n=1126) and females (2080). Values reported as mean±SD with 95% confidence intervals and normal ranges.**

|  | **Males** | | | | **Females** | | | |
| --- | --- | --- | --- | --- | --- | --- | --- | --- |
| **CMR variables** | **Mean ±SD** | **95% CI*** | | **Normal range** | **Mean ±SD** | **95% CI*** | | **Normal range** |
|  | | **Lower limit** | **Upper limit** |  |  | **Lower limit** | **Upper limit** |  |
| LV ejection fraction (%) | 62± 6 | 62 | 62 | 50- 73 | 64± 6 | 64 | 65 | 53- 76 |
| LV stroke volume index to height(ml/m) | 50± 10 | 50 | 51 | 31- 69 | 44± 8 | 43 | 44 | 28- 59 |
| LV end systolic volume index to height(ml/m) | 31± 8 | 31 | 32 | 15- 48 | 24± 6 | 24 | 25 | 12- 36 |
| LV end diastolic volume index to height (ml/m) | 82± 15 | 81 | 83 | 52-112 | 68± 12 | 67 | 68 | 45- 91 |
| LV myocardial mass systole index to height (g/m) | 67± 12 | 67 | 68 | 44- 91 | 49± 9 | 49 | 50 | 32- 66 |
| LV myocardial mass diastole index to height (g/m) | 64± 12 | 64 | 65 | 41- 87 | 48± 9 | 47 | 48 | 31- 65 |
| LV mass to volume ratio (g/ml) | 0.84±0.16 | 0.83 | 0.85 | 0.53-1.15 | 0.74±0.13 | 0.73 | 0.74 | 0.48-1.00 |
|  |  |  |  |  |  |  |  |  |
| RV ejection fraction (%) | 53± 6 | 53 | 53 | 41- 65 | 58± 6 | 58 | 58 | 46- 70 |
| RV stroke volume index to height (ml/m) | 50± 10 | 50 | 51 | 31- 69 | 43± 8 | 43 | 43 | 28- 59 |
| RV end systolic volume index to height (ml/m) | 45± 12 | 45 | 46 | 21- 69 | 32± 9 | 31 | 32 | 15- 49 |
| RV end diastolic volume index to height (ml/m) | 95± 19 | 94 | 97 | 58-133 | 75± 14 | 74 | 76 | 47-103 |
|  |  |  |  |  |  |  |  |  |
| Min. LA volume, indexed to height (ml/m) | 23± 8 | 23 | 24 | 7- 39 | 20± 7 | 19 | 20 | 7- 33 |
| Max. LA volume, indexed to height (ml/m) | 43± 12 | 42 | 43 | 19- 66 | 38± 10 | 38 | 39 | 19- 57 |
| LA EF (%) | 46± 10 | 46 | 47 | 26- 66 | 49± 11 | 48 | 49 | 28- 70 |
| LA SV, indexed to height (ml/m) | 20± 7 | 19 | 20 | 6- 33 | 19± 6 | 18 | 19 | 7- 31 |

***Study cohort – excluded subjects with history of CVD or with risk factors of CVD –hypertension, diabetes, obesity, smoking or dyslipidemia***

***History of CVD – Aortic stenosis, Atrial fibrillation, Heart failure, Mitral stenosis, Previous PCI, Previous CABG, Valve surgery, TAVI, Hx of myocardial infarction***

**** – CI calculated based on the SE***

***** – P value for testing males vs females***

***Reference ranges were calculated based on the formula mean ± t_0.975,n–1_*sqrt[(n+1)/n]*SD***

***EF, ejection fraction; SV, stroke volume***

| **CMR variables** | **35 ≤ Age < 45** | | **45 ≤ Age < 55** | | **55≤ Age < 65** | | **65 ≤ Age < 75** | |
| --- | --- | --- | --- | --- | --- | --- | --- | --- |
|  | **n =141** | **Normal Range** | **n =408** | **Normal Range** | **n =383** | **Normal Range** | **n =194** | **Normal Range** |
| LV ejection fraction (%) | 61 ± 6 | 49- 73 | 62 ± 6 | 50- 73 | 62 ± 6 | 51- 73 | 62 ± 6 | 50- 75 |
| LV stroke volume (ml) | 93 ± 19 | 55-130 | 92 ± 18 | 56-127 | 89 ± 18 | 54-125 | 81 ± 16 | 49-113 |
| LV end systolic volume (ml) | 60 ± 16 | 28- 91 | 57 ± 15 | 27- 87 | 55 ± 15 | 25- 85 | 50 ± 15 | 21- 79 |
| LV end diastolic volume (ml) | 152 ± 29 | 95-209 | 149 ± 28 | 93-205 | 144 ± 29 | 88-201 | 131 ± 26 | 80-183 |
| LV myocardial mass systole (g) | 121 ± 23 | 75-168 | 121 ± 21 | 79-163 | 120 ± 22 | 76-165 | 110 ± 19 | 74-147 |
| LV myocardial mass diastole (g) | 117 ± 22 | 74-161 | 116 ± 21 | 75-157 | 115 ± 23 | 70-160 | 104 ± 16 | 72-137 |
| LV mass to volume ratio (g/ml) | 0.81±0.13 | 0.54-1.07 | 0.83±0.15 | 0.53-1.13 | 0.85±0.15 | 0.56-1.14 | 0.86±0.20 | 0.47-1.26 |
|  |  |  |  |  |  |  |  |  |
| RV ejection fraction (%) | 51 ± 6 | 39- 64 | 53 ± 6 | 41- 64 | 53 ± 6 | 41- 66 | 54 ± 6 | 41- 66 |
| RV stroke volume (ml) | 92 ± 19 | 54-130 | 91 ± 18 | 56-126 | 89 ± 18 | 54-124 | 81 ± 16 | 49-113 |
| RV end systolic volume (ml) | 89 ± 25 | 39-138 | 83 ± 22 | 40-126 | 79 ± 23 | 34-124 | 71 ± 19 | 33-109 |
| RV end diastolic volume (ml) | 181 ± 39 | 104-258 | 174 ± 35 | 105-243 | 168 ± 36 | 98-238 | 152 ± 30 | 92-212 |
|  |  |  |  |  |  |  |  |  |
| Min. LA volume (ml) | 39 ± 12 | 15- 62 | 40 ± 13 | 15- 66 | 43 ± 15 | 13- 72 | 41 ± 16 | 9- 72 |
| Max. LA volume (ml) | 71 ± 20 | 32-111 | 76 ± 21 | 34-117 | 79 ± 21 | 37-121 | 72 ± 23 | 28-117 |
| LA ejection fraction (%) | 46 ± 10 | 27- 65 | 47 ± 10 | 26- 67 | 46 ± 10 | 27- 65 | 45 ± 11 | 22- 67 |
| LA SV (ml) | 33 ± 11 | 10- 56 | 35 ± 12 | 11- 59 | 36 ± 12 | 13- 60 | 32 ± 11 | 10- 54 |

**Suppl Table 2a: Biventricular and left atrial absolute reference values for males 35 to 75 years, stratified by 10–year age categories. Values reported as mean±SD with normal ranges.**

***Study cohort – excluded subjects with history of CVD or with risk factors of CVD –hypertension, diabetes, obesity, smoking or dyslipidemia***

***History of CVD – Aortic stenosis, Atrial fibrillation, Heart failure, Mitral stenosis, Previous PCI, Previous CABG, Valve surgery, TAVI, Hx of myocardial infarction***

**** – P value for testing if all the means are equal***

***Reference ranges were calculated based on the formulae  mean ± t_0.975,n–1_*sqrt[(n+1)/n]*SD***

**Suppl Table 2b: Biventricular and left atrial reference values indexed to height for males 35 to 75 years, stratified by 10–year age categories. Values reported as mean±SD with normal ranges.**

| **CMR variables** | **35 ≤ Age < 45** | | **45 ≤ Age < 55** | | **55≤ Age < 65** | | **65 ≤ Age < 75** | |
| --- | --- | --- | --- | --- | --- | --- | --- | --- |
|  | **n =141** | **Normal Range** | **n =408** | **Normal Range** | **n =383** | **Normal Range** | **n =194** | **Normal Range** |
| LV ejection fraction (%) | 61 ± 6 | 49- 73 | 62 ± 6 | 50- 73 | 62 ± 6 | 51- 73 | 62 ± 6 | 50- 75 |
| LV stroke volume index to height(ml/m) | 52 ± 10 | 33- 71 | 51 ± 10 | 32- 70 | 51 ± 10 | 32- 70 | 47 ± 9 | 28- 65 |
| LV end systolic volume index to height(ml/m) | 33 ± 9 | 16- 51 | 32 ± 8 | 16- 48 | 31 ± 8 | 15- 48 | 28 ± 8 | 13- 44 |
| LV end diastolic volume index to height (ml/m) | 85 ± 15 | 56-115 | 83 ± 15 | 54-113 | 82 ± 15 | 52-112 | 75 ± 14 | 46-103 |
| LV myocardial mass systole index to height (g/m) | 68 ± 12 | 44- 92 | 68 ± 12 | 45- 91 | 68 ± 12 | 45- 92 | 63 ± 10 | 42- 84 |
| LV myocardial mass diastole index to height (g/m) | 66 ± 11 | 43- 89 | 65 ± 12 | 42- 88 | 65 ± 12 | 41- 89 | 60 ± 9 | 41- 78 |
| LV mass to volume ratio (g/ml) | 0.81 ±0.13 | 0.54-1.07 | 0.83 ±0.15 | 0.53-1.13 | 0.85 ±0.15 | 0.56-1.14 | 0.86 ±0.20 | 0.47-1.26 |
|  |  |  |  |  |  |  |  |  |
| RV ejection fraction (%) | 51 ± 6 | 39- 64 | 53 ± 6 | 41- 64 | 53 ± 6 | 41- 66 | 54 ± 6 | 41- 66 |
| RV stroke volume index to height (ml/m) | 52 ± 10 | 32- 72 | 51 ± 9 | 32- 70 | 51 ± 10 | 32- 70 | 46 ± 9 | 28- 65 |
| RV end systolic volume index to height (ml/m) | 50 ± 14 | 23- 77 | 46 ± 12 | 23- 69 | 45 ± 13 | 20- 70 | 40 ± 11 | 19- 62 |
| RV end diastolic volume index to height (ml/m) | 102 ± 20 | 61-142 | 97 ± 18 | 61-134 | 95 ± 19 | 58-133 | 87 ± 17 | 53-120 |
|  |  |  |  |  |  |  |  |  |
| Min. LA volume, indexed to height (ml/m) | 22 ± 6 | 9- 34 | 23 ± 7 | 8- 37 | 24 ± 9 | 7- 41 | 23 ± 9 | 6- 41 |
| Max. LA volume, indexed to height (ml/m) | 40 ± 11 | 19- 61 | 42 ± 12 | 19- 65 | 45 ± 12 | 21- 68 | 41 ± 13 | 16- 66 |
| LA ejection fraction (%) | 46 ± 10 | 27- 65 | 47 ± 10 | 26- 67 | 46 ± 10 | 27- 65 | 45 ± 11 | 22- 67 |
| LA SV, indexed to height (ml/m) | 19 ± 6 | 6- 31 | 20 ± 7 | 6- 33 | 21 ± 7 | 7- 34 | 18 ± 6 | 6- 31 |

***Study cohort – excluded subjects with history of CVD or with risk factors of CVD –hypertension, diabetes, obesity, smoking or dyslipidemia***

***History of CVD – Aortic stenosis, Atrial fibrillation, Heart failure, Mitral stenosis, Previous PCI, Previous CABG, Valve surgery, TAVI, Hx of myocardial infarction***

**** – P value for testing if all the means are equal***

***Reference ranges were calculated based on the formulae  mean ± t_0.975,n–1_*sqrt[(n+1)/n]*SD***

**Suppl Table 3a: Biventricular and left atrial absolute reference values for females 35 to 75 years, stratified by 10–year age categories. Values reported as mean±SD with normal ranges.**

| **CMR variables** | **35 ≤ Age < 45** | | **45 ≤ Age < 55** | | **55≤ Age < 65** | | **65 ≤ Age < 75** | |
| --- | --- | --- | --- | --- | --- | --- | --- | --- |
|  | **n =228** | **Normal Range** | **n =744** | **Normal Range** | **n =795** | **Normal Range** | **n =313** | **Normal Range** |
| LV ejection fraction (%) | 64 ± 5 | 53- 74 | 64 ± 5 | 54- 75 | 64 ± 6 | 53- 76 | 65 ± 6 | 54- 77 |
| LV stroke volume (ml) | 77 ± 15 | 47-106 | 73 ± 14 | 46-101 | 69 ± 13 | 44- 94 | 67 ± 12 | 43- 92 |
| LV end systolic volume (ml) | 44 ± 11 | 22- 66 | 41 ± 10 | 21- 61 | 39 ± 10 | 18- 59 | 36 ± 10 | 16- 55 |
| LV end diastolic volume (ml) | 120 ± 22 | 76-164 | 115 ± 21 | 74-155 | 108 ± 20 | 69-147 | 103 ± 19 | 67-140 |
| LV myocardial mass systole (g) | 82 ± 15 | 53-111 | 81 ± 15 | 51-111 | 81 ± 15 | 52-109 | 78 ± 16 | 47-108 |
| LV myocardial mass diastole (g) | 81 ± 16 | 49-112 | 79 ± 15 | 50-108 | 78 ± 15 | 49-107 | 74 ± 14 | 46-103 |
| LV mass to volume ratio (g/ml) | 0.69±0.11 | 0.47-0.91 | 0.72±0.12 | 0.47-0.96 | 0.76±0.14 | 0.50-1.03 | 0.76±0.14 | 0.49-1.04 |
|  |  |  |  |  |  |  |  |  |
| RV ejection fraction (%) | 56 ± 6 | 45- 68 | 58 ± 6 | 46- 69 | 58 ± 7 | 45- 71 | 59 ± 6 | 47- 72 |
| RV stroke volume (ml) | 76 ± 15 | 46-105 | 73 ± 14 | 46-100 | 68 ± 13 | 43- 94 | 66 ± 12 | 42- 90 |
| RV end systolic volume (ml) | 60 ± 15 | 29- 90 | 54 ± 15 | 25- 83 | 50 ± 15 | 21- 80 | 46 ± 13 | 21- 71 |
| RV end diastolic volume (ml) | 136 ± 26 | 84-187 | 127 ± 25 | 78-175 | 119 ± 23 | 73-165 | 112 ± 21 | 71-153 |
|  |  |  |  |  |  |  |  |  |
| Min. LA volume (ml) | 28 ± 10 | 9- 48 | 31 ± 10 | 11- 51 | 33 ± 11 | 10- 55 | 34 ± 12 | 11- 58 |
| Max. LA volume (ml) | 59 ± 16 | 28- 90 | 63 ± 16 | 31- 94 | 62 ± 17 | 30- 95 | 64 ± 17 | 30- 98 |
| LA ejection fraction (%) | 52 ± 11 | 30- 73 | 50 ± 10 | 30- 71 | 48 ± 11 | 26- 69 | 47 ± 10 | 27- 67 |
| LA SV (ml) | 31 ± 10 | 11- 51 | 32 ± 10 | 12- 52 | 30 ± 10 | 10- 49 | 30 ± 10 | 10- 50 |

***Study cohort – excluded subjects with history of CVD or with risk factors of CVD –hypertension, diabetes, obesity, smoking or dyslipidemia***

***History of CVD – Aortic stenosis, Atrial fibrillation, Heart failure, Mitral stenosis, Previous PCI, Previous CABG, Valve surgery, TAVI, Hx of myocardial infarction***

**** – P value for testing if all the means are equal***

***Reference ranges were calculated based on the formulae  mean ± t_0.975,n–1_*sqrt[(n+1)/n]*SD***

**Suppl Table 3b: Biventricular and left atrial reference values indexed to height for females 35 to 75 years, stratified by 10–year age categories. Values reported as mean±SD with normal ranges.**

| **CMR variables** | **35 ≤ Age < 45** | | **45 ≤ Age < 55** | | **55≤ Age < 65** | | **65 ≤ Age < 75** | |
| --- | --- | --- | --- | --- | --- | --- | --- | --- |
|  | **n =228** | **Normal Range** | **n =744** | **Normal Range** | **n =795** | **Normal Range** | **n =313** | **Normal Range** |
| LV ejection fraction (%) | 64 ± 5 | 53- 74 | 64 ± 5 | 54- 75 | 64 ± 6 | 53- 76 | 65 ± 6 | 54- 77 |
| LV stroke volume index to height(ml/m) | 46 ± 8 | 30- 62 | 45 ± 8 | 29- 61 | 42 ± 7 | 28- 57 | 41 ± 7 | 27- 56 |
| LV end systolic volume index to height(ml/m) | 26 ± 6 | 14- 39 | 25 ± 6 | 13- 37 | 24 ± 6 | 12- 36 | 22 ± 6 | 10- 34 |
| LV end diastolic volume index to height (ml/m) | 73 ± 12 | 49- 96 | 70 ± 12 | 46- 93 | 66 ± 11 | 44- 88 | 63 ± 11 | 42- 85 |
| LV myocardial mass systole index to height (g/m) | 49 ± 8 | 33- 66 | 49 ± 9 | 32- 67 | 50 ± 8 | 33- 66 | 48 ± 9 | 30- 66 |
| LV myocardial mass diastole index to height (g/m) | 49 ± 9 | 31- 67 | 48 ± 9 | 31- 66 | 48 ± 8 | 31- 64 | 46 ± 9 | 29- 63 |
| LV mass to volume ratio (g/ml) | 0.69 ±0.11 | 0.47-0.91 | 0.72 ±0.12 | 0.47-0.96 | 0.76 ±0.14 | 0.50-1.03 | 0.76 ±0.14 | 0.49-1.04 |
|  |  |  |  |  |  |  |  |  |
| RV ejection fraction (%) | 56 ± 6 | 45- 68 | 58 ± 6 | 46- 69 | 58 ± 7 | 45- 71 | 59 ± 6 | 47- 72 |
| RV stroke volume index to height (ml/m) | 46 ± 8 | 30- 62 | 44 ± 8 | 28- 60 | 42 ± 7 | 27- 57 | 41 ± 7 | 26- 55 |
| RV end systolic volume index to height (ml/m) | 36 ± 9 | 19- 53 | 33 ± 9 | 16- 50 | 31 ± 9 | 14- 48 | 28 ± 8 | 14- 43 |
| RV end diastolic volume index to height (ml/m) | 82 ± 14 | 54-110 | 77 ± 14 | 49-106 | 73 ± 13 | 47- 99 | 69 ± 12 | 45- 93 |
|  |  |  |  |  |  |  |  |  |
| Min. LA volume, indexed to height (ml/m) | 17 ± 6 | 6- 28 | 19 ± 6 | 7- 31 | 20 ± 7 | 7- 34 | 21 ± 7 | 7- 36 |
| Max. LA volume, indexed to height (ml/m) | 36 ± 9 | 18- 53 | 38 ± 10 | 19- 57 | 38 ± 10 | 19- 58 | 40 ± 10 | 19- 60 |
| LA ejection fraction (%) | 52 ± 11 | 30- 73 | 50 ± 10 | 30- 71 | 48 ± 11 | 26- 69 | 47 ± 10 | 27- 67 |
| LA SV, indexed to height (ml/m) | 18 ± 6 | 7- 30 | 19 ± 6 | 7- 31 | 18 ± 6 | 6- 30 | 18 ± 6 | 6- 30 |

***Study cohort – excluded subjects with history of CVD or with risk factors of CVD –hypertension, diabetes, obesity, smoking or dyslipidemia***

***History of CVD – Aortic stenosis, Atrial fibrillation, Heart failure, Mitral stenosis, Previous PCI, Previous CABG, Valve surgery, TAVI, Hx of myocardial infarction***

**** – P value for testing if all the means are equal***

***Reference ranges were calculated based on the formula mean ± t_0.975,n–1_*sqrt[(n+1)/n]*SD***

**Suppl Table 4a: Biventricular and atrial absolute reference values for healthy males (n=861) and females (1604) for white Caucasians only. Values reported as mean±SD with 95% confidence intervals and normal ranges.**

|  | **Males** | | | | **Females** | | | |
| --- | --- | --- | --- | --- | --- | --- | --- | --- |
| **CMR variables** | **Mean ±SD** | **95% CI*** | | **Normal range** | **Mean ±SD** | **95% CI*** | | **Normal range** |
|  | | **Lower limit** | **Upper limit** |  |  | **Lower limit** | **Upper limit** |  |
| LV ejection fraction (%) | 62 ± 6 | 61 | 62 | 50- 74 | 64 ± 6 | 64 | 64 | 53- 75 |
| LV stroke volume (ml) | 93 ± 17 | 92 | 94 | 59-127 | 73 ± 14 | 73 | 74 | 47-100 |
| LV end systolic volume (ml) | 58 ± 15 | 57 | 59 | 28- 88 | 41 ± 10 | 41 | 42 | 21- 62 |
| LV end diastolic volume (ml) | 151 ± 28 | 149 | 153 | 97-205 | 115 ± 20 | 114 | 116 | 75-154 |
| LV myocardial mass systole (g) | 122 ± 22 | 121 | 123 | 80-164 | 82 ± 15 | 82 | 83 | 53-112 |
| LV myocardial mass diastole (g) | 116 ± 21 | 115 | 118 | 75-158 | 79 ± 15 | 79 | 80 | 50-109 |
| LV mass to volume ratio (g/ml) | 0.82 ±0.15 | 0.81 | 0.83 | 0.53-1.12 | 0.73 ±0.13 | 0.72 | 0.74 | 0.47-0.98 |
|  |  |  |  |  |  |  |  |  |
| RV ejection fraction (%) | 53 ± 6 | 53 | 54 | 41- 66 | 58 ± 6 | 58 | 58 | 46- 70 |
| RV stroke volume (ml) | 92 ± 17 | 91 | 94 | 58-127 | 72 ± 14 | 72 | 73 | 46- 99 |
| RV end systolic volume (ml) | 83 ± 23 | 81 | 84 | 37-129 | 53 ± 15 | 52 | 54 | 24- 83 |
| RV end diastolic volume (ml) | 175 ± 35 | 173 | 178 | 106-245 | 126 ± 25 | 124 | 127 | 77-174 |
|  |  |  |  |  |  |  |  |  |
| Min. LA volume (ml) | 61 ± 10 | 60 | 61 | 42- 79 | 63 ± 9 | 62 | 63 | 45- 80 |
| Max. LA volume (ml) | 42 ± 15 | 41 | 43 | 14- 71 | 33 ± 11 | 32 | 33 | 11- 54 |
| LA ejection fraction (%) | 78 ± 21 | 77 | 80 | 36-120 | 64 ± 17 | 63 | 65 | 32- 97 |
| LA SV (ml) | 47 ± 10 | 46 | 47 | 27- 67 | 50 ± 11 | 49 | 50 | 29- 71 |

***Healthy cohort – excluded subjects with history of CVD or with risk factors of CVD –hypertension, diabetes, obesity, smoking or dyslipidemia***

***History of CVD – Aortic stenosis, Atrial fibrillation, Heart failure, Mitral stenosis, Previous PCI, Previous CABG, Valve surgery, TAVI, Hx of myocardial infarction***

**** – CI calculated based on the SE***

***** – P value for testing males vs females***

***Reference ranges were calculated based on the formule mean ± t_0.975,n–1_*sqrt[(n+1)/n]*SD***

**Suppl Table 4b: Biventricular and left atrial reference values index to height for healthy males (n=861) and females (1604) for white Caucasians only. Values reported as mean±SD with 95% confidence intervals and normal ranges.**

|  | **Males** | | | | **Females** | | | |
| --- | --- | --- | --- | --- | --- | --- | --- | --- |
| **CMR variables** | **Mean ±SD** | **95% CI*** | | **Normal range** | **Mean ±SD** | **95% CI*** | | **Normal range** |
|  | | **Lower limit** | **Upper limit** |  |  | **Lower limit** | **Upper limit** |  |
| LV ejection fraction (%) | 62 ± 6 | 61 | 62 | 50- 74 | 64 ± 6 | 64 | 64 | 53- 75 |
| LV stroke volume index to height(ml/m) | 52 ± 9 | 52 | 53 | 34- 71 | 45 ± 8 | 44 | 45 | 29- 60 |
| LV end systolic volume index to height(ml/m) | 32 ± 8 | 32 | 33 | 16- 49 | 25 ± 6 | 25 | 25 | 13- 37 |
| LV end diastolic volume index to height (ml/m) | 85 ± 15 | 84 | 86 | 56-114 | 70 ± 11 | 69 | 70 | 47- 92 |
| LV myocardial mass systole index to height (g/m) | 68 ± 12 | 68 | 69 | 45- 92 | 50 ± 9 | 50 | 50 | 33- 67 |
| LV myocardial mass diastole index to height (g/m) | 65 ± 12 | 65 | 66 | 42- 88 | 48 ± 9 | 48 | 49 | 31- 65 |
| LV mass to volume ratio (g/ml) | 0.82 ±0.15 | 0.81 | 0.83 | 0.53-1.12 | 0.73 ±0.13 | 0.72 | 0.74 | 0.47-0.98 |
|  |  |  |  |  |  |  |  |  |
| RV ejection fraction (%) | 53 ± 6 | 53 | 54 | 41- 66 | 58 ± 6 | 58 | 58 | 46- 70 |
| RV stroke volume index to height (ml/m) | 52 ± 9 | 51 | 53 | 33- 70 | 44 ± 8 | 44 | 44 | 29- 59 |
| RV end systolic volume index to height (ml/m) | 46 ± 13 | 45 | 47 | 22- 71 | 32 ± 9 | 32 | 33 | 15- 49 |
| RV end diastolic volume index to height (ml/m) | 98 ± 19 | 97 | 99 | 61-135 | 76 ± 14 | 76 | 77 | 49-104 |
|  |  |  |  |  |  |  |  |  |
| Min. LA volume, indexed to height (ml/m) | 24 ± 8 | 23 | 24 | 8- 40 | 20 ± 7 | 19 | 20 | 7- 33 |
| Max. LA volume, indexed to height (ml/m) | 44 ± 12 | 43 | 45 | 21- 68 | 39 ± 10 | 39 | 40 | 20- 58 |
| LA ejection fraction (%) | 47 ± 10 | 46 | 47 | 27- 67 | 50 ± 11 | 49 | 50 | 29- 71 |
| LA SV, indexed to height (ml/m) | 20 ± 7 | 20 | 21 | 7- 34 | 19 ± 6 | 19 | 20 | 7- 31 |

***Healthy cohort – excluded subjects with history of CVD or with risk factors of CVD –hypertension, diabetes, obesity, smoking or dyslipidemia***

***History of CVD – Aortic stenosis, Atrial fibrillation, Heart failure, Mitral stenosis, Previous PCI, Previous CABG, Valve surgery, TAVI, Hx of myocardial infarction***

**** – CI calculated based on the SE***

***** – P value for testing males vs females***

***Reference ranges were calculated based on the formule mean ± t_0.975,n–1_*sqrt[(n+1)/n]*SD***

**Suppl Table 4c: Biventricular and atrial reference values indexed to BSA for healthy males (n=861) and females (1604) for white Caucasians only. Values reported as mean±SD with 95% confidence intervals and normal ranges.**

|  | **Males** | | | | **Females** | | | |
| --- | --- | --- | --- | --- | --- | --- | --- | --- |
| **CMR variables** | **Mean ±SD** | **95% CI*** | | **Normal range** | **Mean ±SD** | **95% CI*** | | **Normal range** |
|  | | **Lower limit** | **Upper limit** |  |  | **Lower limit** | **Upper limit** |  |
| LV ejection fraction (%) | 62 ± 6 | 61 | 62 | 50- 74 | 64 ± 6 | 64 | 64 | 53- 75 |
| LV stroke volume indexed to BSA (ml/m^2^) | 47 ± 8 | 46 | 47 | 31- 63 | 43 ± 7 | 42 | 43 | 29- 57 |
| LV end-systolic volume indexed to BSA (ml/m^2^) | 29 ± 7 | 29 | 30 | 15- 44 | 24 ± 6 | 24 | 24 | 13- 35 |
| LV end-diastolic volume indexed to BSA (ml/m^2^) | 76 ± 13 | 75 | 77 | 51-101 | 67 ± 10 | 66 | 67 | 46- 87 |
| LV mass (systole), indexed to BSA (g/m^2^) | 62 ± 10 | 61 | 62 | 42- 81 | 48 ± 8 | 48 | 48 | 33- 63 |
| LV mass (diastole), indexed to BSA (g/m^2^) | 59 ± 10 | 58 | 59 | 39- 78 | 46 ± 8 | 46 | 47 | 31- 62 |
| LV mass to volume ratio (g/ml) | 0.82 ±0.15 | 0.81 | 0.83 | 0.53-1.12 | 0.73 ±0.13 | 0.72 | 0.74 | 0.47-0.98 |
|  |  |  |  |  |  |  |  |  |
| RV ejection fraction (%) | 53 ± 6 | 53 | 54 | 41- 66 | 58 ± 6 | 58 | 58 | 46- 70 |
| RV stroke volume indexed to BSA (ml/m^2^) | 47 ± 8 | 46 | 47 | 30- 63 | 42 ± 7 | 42 | 43 | 28- 56 |
| RV end-diastolic volume indexed to BSA (ml/m^2^) | 88 ± 17 | 87 | 90 | 56-121 | 73 ± 13 | 72 | 74 | 48- 99 |
| RV end-systolic volume indexed to BSA (ml/m^2^) | 42 ± 11 | 41 | 42 | 20- 64 | 31 ± 8 | 30 | 31 | 14- 47 |
|  |  |  |  |  |  |  |  |  |
| Min. LA volume, indexed to BSA(ml/m^2^) | 21 ± 7 | 21 | 22 | 7- 36 | 19 ± 6 | 19 | 19 | 7- 31 |
| Max. LA volume, indexed to BSA(ml/m^2^) | 40 ± 11 | 39 | 40 | 19- 60 | 37 ± 9 | 37 | 38 | 19- 55 |
| LA ejection fraction (%) | 47 ± 10 | 46 | 47 | 27- 67 | 50 ± 11 | 49 | 50 | 29- 71 |
| LA SV, indexed to BSA(ml/m^2^) | 18 ± 6 | 18 | 19 | 7- 30 | 18 ± 6 | 18 | 19 | 7- 30 |

***Healthy cohort – excluded subjects with history of CVD or with risk factors of CVD –hypertension, diabetes, obesity, smoking or dyslipidemia***

***History of CVD – Aortic stenosis, Atrial fibrillation, Heart failure, Mitral stenosis, Previous PCI, Previous CABG, Valve surgery, TAVI, Hx of myocardial infarction***

**** – CI calculated based on the SE***

***** – P value for testing males vs females***

***Reference ranges were calculated based on the formule mean ± t_0.975,n–1_*sqrt[(n+1)/n]*SD***

**Suppl Table 5a: Biventricular and left atrial absolute reference values for healthy males (n=191) and females (356) for Chinese only. Values reported as mean±SD with 95% confidence intervals and normal ranges.**

|  | **Males** | | | | **Females** | | | |
| --- | --- | --- | --- | --- | --- | --- | --- | --- |
| **CMR variables** | **Mean ±SD** | **95% CI*** | | **Normal range** | **Mean ±SD** | **95% CI*** | | **Normal range** |
|  | | **Lower limit** | **Upper limit** |  |  | **Lower limit** | **Upper limit** |  |
| LV ejection fraction (%) | 62 ± 6 | 61 | 63 | 51- 73 | 66 ± 5 | 65 | 66 | 55- 76 |
| LV stroke volume (ml) | 79 ± 15 | 77 | 81 | 50-108 | 64 ± 11 | 63 | 65 | 43- 85 |
| LV end systolic volume (ml) | 48 ± 13 | 47 | 50 | 23- 74 | 34 ± 9 | 33 | 35 | 16- 51 |
| LV end diastolic volume (ml) | 127 ± 24 | 124 | 130 | 81-174 | 97 ± 17 | 96 | 99 | 65-130 |
| LV myocardial mass systole (g) | 111 ± 19 | 108 | 114 | 74-148 | 74 ± 12 | 72 | 75 | 49- 98 |
| LV myocardial mass diastole (g) | 108 ± 19 | 105 | 110 | 69-146 | 73 ± 13 | 72 | 75 | 48- 98 |
| LV mass to volume ratio (g/ml) | 0.89 ±0.17 | 0.87 | 0.91 | 0.55-1.23 | 0.77 ±0.14 | 0.75 | 0.78 | 0.50-1.04 |
|  |  |  |  |  |  |  |  |  |
| RV ejection fraction (%) | 52 ± 5 | 51 | 53 | 41- 63 | 57 ± 6 | 57 | 58 | 45- 69 |
| RV stroke volume (ml) | 78 ± 14 | 76 | 80 | 50-107 | 63 ± 11 | 62 | 64 | 41- 86 |
| RV end systolic volume (ml) | 73 ± 18 | 70 | 76 | 37-109 | 48 ± 14 | 47 | 50 | 21- 75 |
| RV end diastolic volume (ml) | 151 ± 28 | 147 | 155 | 95-207 | 111 ± 22 | 109 | 114 | 68-155 |
|  |  |  |  |  |  |  |  |  |
| Min. LA volume (ml) | 38 ± 12 | 36 | 39 | 14- 61 | 30 ± 10 | 29 | 31 | 10- 49 |
| Max. LA volume (ml) | 68 ± 17 | 65 | 70 | 34-101 | 56 ± 13 | 54 | 57 | 29- 82 |
| LA ejection fraction (%) | 45 ± 10 | 43 | 46 | 24- 65 | 47 ± 10 | 46 | 48 | 27- 67 |
| LA SV (ml) | 30 ± 10 | 29 | 32 | 10- 50 | 26 ± 8 | 25 | 27 | 10- 42 |

***Healthy cohort – excluded subjects with history of CVD or with risk factors of CVD –hypertension, diabetes, obesity, smoking or dyslipidemia***

***History of CVD – Aortic stenosis, Atrial fibrillation, Heart failure, Mitral stenosis, Previous PCI, Previous CABG, Valve surgery, TAVI, Hx of myocardial infarction***

**** – CI calculated based on the SE***

***** – P value for testing males vs females***

***Reference ranges were calculated based on the formule mean ± t_0.975,n–1_*sqrt[(n+1)/n]*SD***

**Suppl Table 5b: Biventricular and left atrial reference values indexed to height for healthy males (n=193) and females (356) for Chinese only. Values reported as mean±SD with 95% confidence intervals and normal ranges.**

|  | **Males** | | | | **Females** | | | |
| --- | --- | --- | --- | --- | --- | --- | --- | --- |
| **CMR variables** | **Mean ±SD** | **95% CI*** | | **Normal range** | **Mean ±SD** | **95% CI*** | | **Normal range** |
|  | | **Lower limit** | **Upper limit** |  |  | **Lower limit** | **Upper limit** |  |
| LV ejection fraction (%) | 62 ± 6 | 61 | 63 | 51- 73 | 66 ± 5 | 65 | 66 | 55- 76 |
| LV stroke volume index to height(ml/m) | 46 ± 8 | 45 | 47 | 30- 61 | 40 ± 6 | 39 | 40 | 27- 52 |
| LV end systolic volume index to height(ml/m) | 28 ± 7 | 27 | 29 | 14- 43 | 21 ± 5 | 20 | 22 | 10- 32 |
| LV end diastolic volume index to height (ml/m) | 74 ± 13 | 72 | 76 | 48- 99 | 61 ± 10 | 60 | 62 | 41- 81 |
| LV myocardial mass systole index to height (g/m) | 64 ± 10 | 63 | 66 | 44- 84 | 46 ± 8 | 45 | 47 | 31- 61 |
| LV myocardial mass diastole index to height (g/m) | 62 ± 10 | 61 | 64 | 42- 83 | 46 ± 8 | 45 | 47 | 31- 61 |
| LV mass to volume ratio (g/ml) | 0.89 ±0.17 | 0.87 | 0.91 | 0.55-1.23 | 0.77 ±0.14 | 0.75 | 0.78 | 0.50-1.04 |
|  |  |  |  |  |  |  |  |  |
| RV ejection fraction (%) | 52 ± 5 | 51 | 53 | 41- 63 | 57 ± 6 | 57 | 58 | 45- 69 |
| RV stroke volume index to height (ml/m) | 45 ± 8 | 44 | 47 | 30- 61 | 39 ± 7 | 39 | 40 | 26- 53 |
| RV end systolic volume index to height (ml/m) | 42 ± 10 | 41 | 44 | 22- 63 | 30 ± 9 | 29 | 31 | 13- 47 |
| RV end diastolic volume index to height (ml/m) | 88 ± 16 | 85 | 90 | 57-119 | 70 ± 13 | 68 | 71 | 43- 96 |
|  |  |  |  |  |  |  |  |  |
| Min. LA volume, indexed to height (ml/m) | 22 ± 7 | 21 | 23 | 8- 35 | 19 ± 6 | 18 | 19 | 6- 31 |
| Max. LA volume, indexed to height (ml/m) | 39 ± 10 | 38 | 41 | 20- 59 | 35 ± 8 | 34 | 36 | 18- 51 |
| LA ejection fraction (%) | 45 ± 10 | 43 | 46 | 24- 65 | 47 ± 10 | 46 | 48 | 27- 67 |
| LA SV, indexed to height (ml/m) | 18 ± 6 | 17 | 18 | 6- 29 | 16 ± 5 | 16 | 17 | 6- 26 |

***Healthy cohort – excluded subjects with history of CVD or with risk factors of CVD –hypertension, diabetes, obesity, smoking or dyslipidemia***

***History of CVD – Aortic stenosis, Atrial fibrillation, Heart failure, Mitral stenosis, Previous PCI, Previous CABG, Valve surgery, TAVI, Hx of myocardial infarction***

**** – CI calculated based on the SE***

***** – P value for testing males vs females***

***Reference ranges were calculated based on the formule mean ± t_0.975,n–1_*sqrt[(n+1)/n]*SD***

**Suppl Table 5c: Biventricular and left atrial reference values indexed to BSA for healthy males (n=193) and females (356) for Chinese only. Values reported as mean±SD with 95% confidence intervals and normal ranges.**

|  | **Males** | | | | **Females** | | | |
| --- | --- | --- | --- | --- | --- | --- | --- | --- |
| **CMR variables** | **Mean ±SD** | **95% CI*** | | **Normal range** | **Mean ±SD** | **95% CI*** | | **Normal range** |
|  | | **Lower limit** | **Upper limit** |  |  | **Lower limit** | **Upper limit** |  |
| LV ejection fraction (%) | 62 ± 6 | 61 | 63 | 51- 73 | 66 ± 5 | 65 | 66 | 55- 76 |
| LV stroke volume indexed to BSA (ml/m^2^) | 42 ± 7 | 41 | 43 | 29- 56 | 40 ± 6 | 40 | 41 | 29- 52 |
| LV end-systolic volume indexed to BSA (ml/m^2^) | 26 ± 6 | 25 | 27 | 13- 39 | 21 ± 5 | 21 | 22 | 11- 32 |
| LV end-diastolic volume indexed to BSA (ml/m^2^) | 69 ± 11 | 67 | 70 | 47- 90 | 62 ± 9 | 61 | 63 | 44- 80 |
| LV mass (systole), indexed to BSA (g/m^2^) | 60 ± 8 | 59 | 61 | 44- 75 | 47 ± 7 | 46 | 47 | 34- 60 |
| LV mass (diastole), indexed to BSA (g/m^2^) | 58 ± 8 | 57 | 59 | 42- 74 | 46 ± 7 | 46 | 47 | 33- 60 |
| LV mass to volume ratio (g/ml) | 0.89 ±0.17 | 0.87 | 0.91 | 0.55-1.23 | 0.77 ±0.14 | 0.75 | 0.78 | 0.50-1.04 |
|  |  |  |  |  |  |  |  |  |
| RV ejection fraction (%) | 52 ± 5 | 51 | 53 | 41- 63 | 57 ± 6 | 57 | 58 | 45- 69 |
| RV stroke volume indexed to BSA (ml/m^2^) | 42 ± 7 | 41 | 43 | 29- 55 | 40 ± 7 | 39 | 41 | 27- 53 |
| RV end-diastolic volume indexed to BSA (ml/m^2^) | 82 ± 13 | 80 | 83 | 56-107 | 71 ± 13 | 69 | 72 | 45- 96 |
| RV end-systolic volume indexed to BSA (ml/m^2^) | 39 ± 9 | 38 | 41 | 21- 57 | 31 ± 8 | 30 | 31 | 14- 47 |
|  |  |  |  |  |  |  |  |  |
| Min. LA volume, indexed to BSA(ml/m^2^) | 20 ± 6 | 19 | 21 | 8- 32 | 19 ± 6 | 18 | 19 | 7- 30 |
| Max. LA volume, indexed to BSA(ml/m^2^) | 36 ± 8 | 35 | 38 | 20- 53 | 35 ± 8 | 34 | 36 | 20- 51 |
| LA ejection fraction (%) | 45 ± 10 | 43 | 46 | 24- 65 | 47 ± 10 | 46 | 48 | 27- 67 |
| LA SV, indexed to BSA(ml/m^2^) | 16 ± 5 | 16 | 17 | 6- 26 | 17 ± 5 | 16 | 17 | 7- 26 |

***Healthy cohort – excluded subjects with history of CVD or with risk factors of CVD –hypertension, diabetes, obesity, smoking or dyslipidemia***

***History of CVD – Aortic stenosis, Atrial fibrillation, Heart failure, Mitral stenosis, Previous PCI, Previous CABG, Valve surgery, TAVI, Hx of myocardial infarction***

**** – CI calculated based on the SE***

***** – P value for testing males vs females***

***Reference ranges were calculated based on the formule mean ± t_0.975,n–1_*sqrt[(n+1)/n]*SD***

**Suppl Table 6a: Biventricular and left atrial absolute reference values for healthy males (n=53) and females (70) for South Asians only. Values reported as mean±SD with 95% confidence intervals and normal ranges.**

|  | **Males** | | | | **Females** | | | |
| --- | --- | --- | --- | --- | --- | --- | --- | --- |
| **CMR variables** | **Mean ±SD** | **95% CI*** | | **Normal range** | **Mean ±SD** | **95% CI*** | | **Normal range** |
|  | | **Lower limit** | **Upper limit** |  |  | **Lower limit** | **Upper limit** |  |
| LV ejection fraction (%) | 62 ± 5 | 61 | 64 | 51- 73 | 64 ± 5 | 62 | 65 | 53- 75 |
| LV stroke volume (ml) | 72 ± 16 | 67 | 76 | 39-105 | 61 ± 12 | 58 | 64 | 38- 84 |
| LV end systolic volume (ml) | 44 ± 12 | 41 | 47 | 20- 68 | 35 ± 10 | 33 | 38 | 16- 55 |
| LV end diastolic volume (ml) | 116 ± 25 | 109 | 123 | 64-167 | 96 ± 19 | 92 | 101 | 59-134 |
| LV myocardial mass systole (g) | 102 ± 22 | 96 | 108 | 58-145 | 70 ± 13 | 67 | 74 | 44- 96 |
| LV myocardial mass diastole (g) | 99 ± 19 | 94 | 104 | 61-137 | 70 ± 14 | 67 | 73 | 42- 97 |
| LV mass to volume ratio (g/ml) | 0.89 ±0.19 | 0.84 | 0.95 | 0.52-1.27 | 0.75 ±0.14 | 0.71 | 0.78 | 0.46-1.03 |
|  |  |  |  |  |  |  |  |  |
| RV ejection fraction (%) | 52 ± 6 | 50 | 53 | 40- 63 | 56 ± 7 | 55 | 58 | 42- 70 |
| RV stroke volume (ml) | 72 ± 17 | 67 | 77 | 38-106 | 61 ± 11 | 58 | 63 | 38- 84 |
| RV end systolic volume (ml) | 68 ± 21 | 62 | 74 | 26-111 | 48 ± 14 | 45 | 51 | 20- 76 |
| RV end diastolic volume (ml) | 140 ± 35 | 131 | 150 | 70-210 | 109 ± 21 | 103 | 114 | 65-152 |
|  |  |  |  |  |  |  |  |  |
| Min. LA volume (ml) | 33 ± 11 | 31 | 36 | 12- 55 | 30 ± 11 | 28 | 33 | 9- 52 |
| Max. LA volume (ml) | 59 ± 20 | 54 | 65 | 18-101 | 56 ± 16 | 52 | 60 | 23- 89 |
| LA ejection fraction (%) | 45 ± 11 | 42 | 48 | 23- 68 | 45 ± 11 | 43 | 48 | 22- 68 |
| LA SV (ml) | 27 ± 11 | 24 | 30 | 5- 49 | 25 ± 10 | 23 | 28 | 6- 45 |

***Healthy cohort – excluded subjects with history of CVD or with risk factors of CVD –hypertension, diabetes, obesity, smoking or dyslipidemia***

***History of CVD – Aortic stenosis, Atrial fibrillation, Heart failure, Mitral stenosis, Previous PCI, Previous CABG, Valve surgery, TAVI, Hx of myocardial infarction***

**** – CI calculated based on the SE***

***** – P value for testing males vs females***

***Reference ranges were calculated based on the formule mean ± t_0.975,n–1_*sqrt[(n+1)/n]*SD***

**Suppl Table 6b: Biventricular and atrial reference values indexed to height for healthy males (n=53) and females (70) for South Asians only. Values reported as mean±SD with 95% confidence intervals and normal ranges.**

|  | **Males** | | | | **Females** | | | |
| --- | --- | --- | --- | --- | --- | --- | --- | --- |
| **CMR variables** | **Mean ±SD** | **95% CI*** | | **Normal range** | **Mean ±SD** | **95% CI*** | | **Normal range** |
|  | | **Lower limit** | **Upper limit** |  |  | **Lower limit** | **Upper limit** |  |
| LV ejection fraction (%) | 62 ± 5 | 61 | 64 | 51- 73 | 64 ± 5 | 62 | 65 | 53- 75 |
| LV stroke volume index to height(ml/m) | 41 ± 9 | 38 | 43 | 22- 59 | 39 ± 10 | 37 | 41 | 20- 58 |
| LV end systolic volume index to height(ml/m) | 25 ± 7 | 23 | 27 | 12- 39 | 22 ± 6 | 21 | 24 | 10- 34 |
| LV end diastolic volume index to height (ml/m) | 66 ± 14 | 62 | 70 | 37- 94 | 61 ± 14 | 58 | 65 | 34- 89 |
| LV myocardial mass systole index to height (g/m) | 58 ± 12 | 54 | 61 | 33- 83 | 45 ± 11 | 42 | 48 | 23- 67 |
| LV myocardial mass diastole index to height (g/m) | 56 ± 11 | 53 | 59 | 35- 78 | 45 ± 11 | 42 | 47 | 23- 66 |
| LV mass to volume ratio (g/ml) | 0.89 ±0.19 | 0.84 | 0.95 | 0.52-1.27 | 0.75 ±0.14 | 0.71 | 0.78 | 0.46-1.03 |
|  |  |  |  |  |  |  |  |  |
| RV ejection fraction (%) | 52 ± 6 | 50 | 53 | 40- 63 | 56 ± 7 | 55 | 58 | 42- 70 |
| RV stroke volume index to height (ml/m) | 41 ± 9 | 38 | 43 | 22- 60 | 39 ± 9 | 36 | 41 | 20- 57 |
| RV end systolic volume index to height (ml/m) | 39 ± 12 | 35 | 42 | 15- 62 | 31 ± 9 | 28 | 33 | 12- 50 |
| RV end diastolic volume index to height (ml/m) | 79 ± 19 | 74 | 85 | 41-118 | 69 ± 16 | 65 | 73 | 36-102 |
|  |  |  |  |  |  |  |  |  |
| Min. LA volume, indexed to height (ml/m) | 19 ± 6 | 17 | 21 | 7- 31 | 19 ± 7 | 18 | 21 | 6- 32 |
| Max. LA volume, indexed to height (ml/m) | 34 ± 12 | 30 | 37 | 10- 57 | 36 ± 11 | 33 | 38 | 14- 57 |
| LA ejection fraction (%) | 45 ± 11 | 42 | 48 | 23- 68 | 45 ± 11 | 43 | 48 | 22- 68 |
| LA SV, indexed to height (ml/m) | 15 ± 6 | 14 | 17 | 3- 28 | 16 ± 7 | 15 | 18 | 3- 30 |

***Healthy cohort – excluded subjects with history of CVD or with risk factors of CVD –hypertension, diabetes, obesity, smoking or dyslipidemia***

***History of CVD – Aortic stenosis, Atrial fibrillation, Heart failure, Mitral stenosis, Previous PCI, Previous CABG, Valve surgery, TAVI, Hx of myocardial infarction***

**** – CI calculated based on the SE***

***** – P value for testing males vs females***

***Reference ranges were calculated based on the formule mean ± t_0.975,n–1_*sqrt[(n+1)/n]*SD***

**Suppl Table 6c: Biventricular and atrial reference values indexed to BSA for healthy males (n=53) and females (70) for South Asians only. Values reported as mean±SD with 95% confidence intervals and normal ranges.**

|  | **Males** | | | | **Females** | | | |
| --- | --- | --- | --- | --- | --- | --- | --- | --- |
| **CMR variables** | **Mean ±SD** | **95% CI*** | | **Normal range** | **Mean ±SD** | **95% CI*** | | **Normal range** |
|  | | **Lower limit** | **Upper limit** |  |  | **Lower limit** | **Upper limit** |  |
| LV ejection fraction (%) | 62 ± 5 | 61 | 64 | 51- 73 | 64 ± 5 | 62 | 65 | 53- 75 |
| LV stroke volume indexed to BSA (ml/m^2^) | 36 ± 8 | 34 | 39 | 21- 52 | 36 ± 7 | 34 | 38 | 22- 50 |
| LV end-systolic volume indexed to BSA (ml/m^2^) | 22 ± 5 | 21 | 24 | 12- 33 | 21 ± 6 | 19 | 22 | 9- 32 |
| LV end-diastolic volume indexed to BSA (ml/m^2^) | 59 ± 11 | 56 | 62 | 36- 82 | 57 ± 11 | 54 | 59 | 34- 79 |
| LV mass (systole), indexed to BSA (g/m^2^) | 52 ± 11 | 49 | 55 | 30- 73 | 41 ± 7 | 40 | 43 | 27- 56 |
| LV mass (diastole), indexed to BSA (g/m^2^) | 50 ± 9 | 48 | 53 | 32- 69 | 41 ± 8 | 39 | 43 | 25- 57 |
| LV mass to volume ratio (g/ml) | 0.89 ±0.19 | 0.84 | 0.95 | 0.52-1.27 | 0.75 ±0.14 | 0.71 | 0.78 | 0.46-1.03 |
|  |  |  |  |  |  |  |  |  |
| RV ejection fraction (%) | 52 ± 6 | 50 | 53 | 40- 63 | 56 ± 7 | 55 | 58 | 42- 70 |
| RV stroke volume indexed to BSA (ml/m^2^) | 36 ± 8 | 34 | 39 | 21- 52 | 36 ± 7 | 34 | 37 | 21- 50 |
| RV end-diastolic volume indexed to BSA (ml/m^2^) | 71 ± 15 | 67 | 75 | 41-100 | 64 ± 12 | 61 | 67 | 39- 89 |
| RV end-systolic volume indexed to BSA (ml/m^2^) | 34 ± 9 | 32 | 37 | 16- 52 | 28 ± 8 | 26 | 30 | 12- 44 |
|  |  |  |  |  |  |  |  |  |
| Min. LA volume, indexed to BSA(ml/m^2^) | 17 ± 5 | 15 | 18 | 6- 28 | 18 ± 6 | 16 | 19 | 6- 30 |
| Max. LA volume, indexed to BSA(ml/m^2^) | 30 ± 10 | 27 | 33 | 10- 50 | 33 ± 9 | 31 | 35 | 14- 51 |
| LA ejection fraction (%) | 45 ± 11 | 42 | 48 | 23- 68 | 45 ± 11 | 43 | 48 | 22- 68 |
| LA SV, indexed to BSA(ml/m^2^) | 14 ± 5 | 12 | 15 | 3- 25 | 15 ± 6 | 14 | 16 | 4- 27 |

***Healthy cohort – excluded subjects with history of CVD or with risk factors of CVD –hypertension, diabetes, obesity, smoking or dyslipidemia***

***History of CVD – Aortic stenosis, Atrial fibrillation, Heart failure, Mitral stenosis, Previous PCI, Previous CABG, Valve surgery, TAVI, Hx of myocardial infarction***

**** – CI calculated based on the SE***

***** – P value for testing males vs females***

***Reference ranges were calculated based on the formule mean ± t_0.975,n–1_*sqrt[(n+1)/n]*SD***
